# Ubiquilin 1 Promotes IFN-γ-Induced Xenophagy of *Mycobacterium tuberculosis*

**DOI:** 10.1371/journal.ppat.1005076

**Published:** 2015-07-30

**Authors:** Erik T. Sakowski, Stefan Koster, Cynthia Portal Celhay, Heidi S. Park, Elina Shrestha, Stefanie E. Hetzenecker, Katie Maurer, Ken Cadwell, Jennifer A. Philips

**Affiliations:** 1 Division of Infectious Diseases and Immunology, Department of Medicine, New York University School of Medicine, New York, New York, United States of America; 2 Department of Microbiology, New York University School of Medicine, New York, New York, United States of America; 3 Kimmel Center for Biology and Medicine at the Skirball Institute of Biomolecular Medicine, New York University School of Medicine, New York, New York, United States of America; 4 Department of Pathology, New York University School of Medicine, New York, New York, United States of America; Portland VA Medical Center, Oregon Health and Science University, UNITED STATES

## Abstract

The success of *Mycobacterium tuberculosis* (Mtb) as a pathogen rests upon its ability to grow intracellularly in macrophages. Interferon-gamma (IFN-γ) is critical in host defense against Mtb and stimulates macrophage clearance of Mtb through an autophagy pathway. Here we show that the host protein ubiquilin 1 (UBQLN1) promotes IFN-γ-mediated autophagic clearance of Mtb. Ubiquilin family members have previously been shown to recognize proteins that aggregate in neurodegenerative disorders. We find that UBQLN1 can interact with Mtb surface proteins and associates with the bacilli *in vitro*. In IFN-γ activated macrophages, UBQLN1 co-localizes with Mtb and promotes the anti-mycobacterial activity of IFN-γ. The association of UBQLN1 with Mtb depends upon the secreted bacterial protein, EsxA, which is involved in permeabilizing host phagosomes. In autophagy-deficient macrophages, UBQLN1 accumulates around Mtb, consistent with the idea that it marks bacilli that traffic through the autophagy pathway. Moreover, UBQLN1 promotes ubiquitin, p62, and LC3 accumulation around Mtb, acting independently of the E3 ligase parkin. In summary, we propose a model in which UBQLN1 recognizes Mtb and in turn recruits the autophagy machinery thereby promoting intracellular control of Mtb. Thus, polymorphisms in ubiquilins, which are known to influence susceptibility to neurodegenerative illnesses, might also play a role in host defense against Mtb.

## Author Summary

More people die from *Mycobacterium tuberculosis* (Mtb), the causative agent of tuberculosis (TB), than any other bacterial pathogen. It has long been appreciated that Mtb can survive and divide within macrophages, white blood cells that normally kill bacteria. Macrophages are able to partially control Mtb through a degradative process called autophagy. Autophagy is activated by the cytokine interferon-gamma (IFN-γ), which promotes control of Mtb infection. How the tubercle bacilli are targeted to the autophagy pathway remains unclear. Here we show that the human protein ubiquilin 1 can interact with Mtb surface proteins and associate with Mtb that are present in the host cell cytosol. We propose a model in which activating autophagy with IFN-γ promotes UBQLN1 recruitment to Mtb, which in turn leads to recruitment of the autophagy machinery, autophagy-mediated degradation of the bacteria, and activation of effector T cells. Since IFN-γ is critical in human control of Mtb, our study suggests that polymorphisms in ubiquilins, known to influence susceptibility to neurodegenerative illnesses, might also play a role in host defense against Mtb.

## Introduction

*Mycobacterium tuberculosis* (Mtb) infects one-third of the world’s population. It can remain dormant in its host for decades and ultimately kills more people than any other bacteria. Mtb survives within macrophages by preventing its own delivery to the degradative, phagolysosomal compartment [1]. Macrophages that are activated by IFN-γ partially overcome the arrest in phagosome maturation imposed by Mtb [2,3]. IFN-γ stimulates macroautophagy [4–6] (hereafter autophagy), a process by which double-membrane organelles termed autophagosomes capture and degrade cytoplasmic components. In non-selective autophagy, which occurs in response to nutrient limitation, a portion of the cytoplasm is engulfed. In a form of autophagy that is called xenophagy, invading microorganisms are targeted. Autophagosomes that sequester Mtb fuse with lysosomes and impair mycobacterial replication. Autophagy partially restricts Mtb replication, and conditions that activate autophagy, including exposure to IFN-γ, promote mycobacterial clearance [4,6–8].

A prevailing model for how autophagy contributes to antimicrobial host defense begins with bacteria damaging or escaping phagosomes [9,10]. In the case of Mtb, damage and/or escape depends upon the mycobacterial ESX-1 Type VII secretion system and the secreted effector EsxA (also known as ESAT-6) [11–15]. Phagosomal damage allows mycobacterial DNA and peptidoglycan to activate host cytosolic sensors [14,16]. Ubiquitinated proteins accumulate around the bacteria, which partially depends upon the E3 ligase parkin (encoded by the *PARK2* gene) [17]. The host proteins p62 (also known as SQSTM1), NDP52 (nuclear dot protein 52 kD, also known as CALCOCO2), and NBR1 (next to BRCA1 gene 1) can all bind ubiquitin as well as the autophagy protein LC3; they are thought to serve as cargo adaptors that link ubiquitin-conjugated Mtb or phagosomal remnants to LC3 [8,9,17]. A major unanswered question in the field is how Mtb, or any other bacteria, are physically linked to the autophagy machinery. In mitophagy, an analogous process in which mitochondria are selectively cleared from the cytoplasm, certain outer mitochondrial membrane proteins directly bind LC3 or recruit parkin to damaged mitochondria [18]. How parkin and other E3 ligases are recruited to the invading tubercle bacilli is uncertain; no mechanism comparable to that described for mitophagy has been shown to link the bacterial surface to the autophagy machinery.

**Fig 1:**
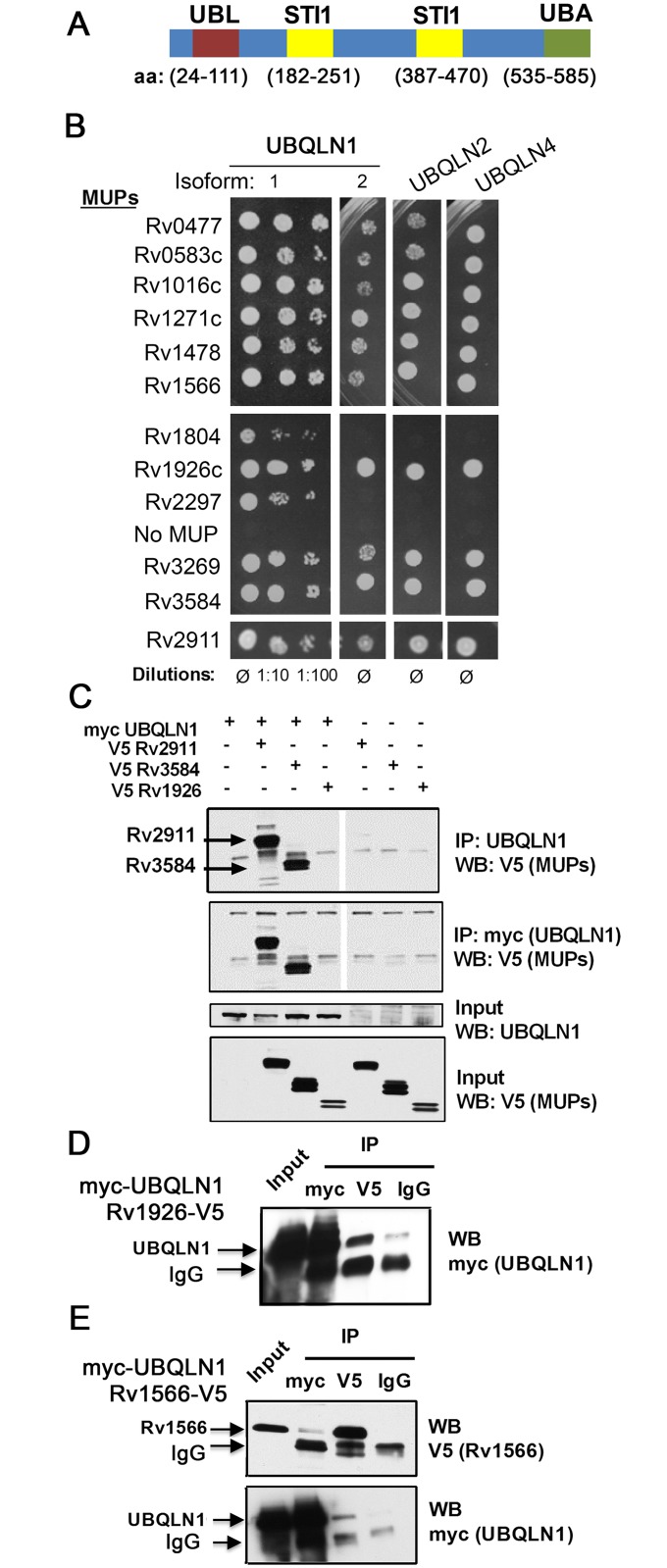
MUPs bind ubiquilins. (A) Domain structure of human UBQLN1 (Interpro: http://www.ebi.ac.uk/interpro/). (B) Gal4 DNA-binding domain fusions of MUPs were tested with Gal4 activation domain fusions of ubiquilins (murine) in Y2H. (C) HEK293 cells transfected with indicated MUP-V5, myc-UBQLN1 (human), or empty vector were immunoprecipitated (IP) with anti-myc or anti-UBQLN1 antibodies. Western blot (WB) of IP and lysate were probed as indicated. (D-E) WB of input lysate and IP from HEK293 cells expressing myc-UBQLN1 and Rv1926-V5 (D) or Rv1566-V5 (E). IP was performed using antibodies directed against myc, V5, or isotype control (IgG).

We found that host UBQLN1 (also known as PLIC-1) binds a subset of Mtb secreted proteins and recognizes Mtb during xenophagy. UBQLN1 is a member of a family of highly related proteins that contain a ubiquitin-like (UBL) domain, a ubiquitin-associated domain (UBA), and STI1 motifs that are found in the co-chaperone Sti-1 (also known as HOP) (Fig 1A). UBQLN1 and UBQLN2 are thought to facilitate degradation of ubiquitinated targets by the proteasome [19,20]. More recently, they have also been shown to play a role in autophagy. Ubiquilins associate with autophagosomes, participate in autophagosome formation, and protect against starvation-induced cell death [21,22]. They are implicated in clearing protein aggregates in neurodegenerative disorders, including Alzheimer’s disease, amyotrophic lateral sclerosis (ALS), and Huntington’s disease [23–25]. Here, we show that UBQLN1 recognizes Mtb, acts upstream of ubiquitination, and promotes autophagy-mediated clearance of Mtb. Therefore, we provide evidence that UBQLN1 serves as a link between the bacterial surface and the host autophagy pathway.

## Results

### UBQLN1 interacts with exported Mtb proteins

Previously, we used a stringent yeast two-hybrid (Y2H) system to map Mtb-human protein-protein interactions. UBQLN1 interacted with 12 Mtb proteins, which we call MUPs for **m**ycobacterial **u**biquilin-interacting **p**roteins [26] (S1 Text, S1 Table). UBQLN1 exhibited selectivity in its interactions, as we identified it only 12 times in a screen of 339 secreted Mtb proteins. It did not interact with any of the 60 non-secreted Mtb proteins that were also screened or Antigen 85b, EsxA, and EsxB when directly tested [26]. Specificity was also indicated by the finding that MUPs interacted weakly or not at all with NDP52, an autophagy receptor that, like UBQLN1, contains a ubiquitin-binding domain (S1 Fig). To evaluate whether MUPs interact with other ubiquilin family members, we examined murine UBQLN1, UBQLN2, and UBQLN4, which are 88%, 67%, and 60% identical to human UBQLN1, respectively. We did not test UBQLN3 because its expression is restricted to the testes [27]. Most MUPs interacted with murine UBQLN1 (both splice isoforms), UBQLN2, and UBQLN4 (Fig 1B). The MUPs are largely uncharacterized; there is not a domain common to all of them, although two MUPs contain p60 domains (S1 Table). Most have a predicted signal peptide that targets them for secretion, and the majority have been found in culture filtrate in at least one study, suggesting they are accessible to host interactions. Few MUPs are found exclusively in the culture filtrate; most are also present in the cell membrane or whole cell lysate (S2 Fig)[28]. To conclude, we found that ubiquilin family members can interact with numerous Mtb secreted and surface proteins.

To determine whether MUPs interact with UBQLN1 in mammalian cells, we expressed V5-tagged MUPs in HEK293 cells. We could detect expression of eight MUPs in HEK293 cells, and we tested four in co-immunoprecipitation assays with human UBQLN1. We could not detect endogenous UBQLN1 in HEK293 cells, so we co-transfected myc-UBQLN1 along with V5-tagged MUPs. myc-UBQLN1 immunoprecipitated MUPs Rv2911 and Rv3584, but not Rv1926. MUPs were not detected in immunoprecipitates from cells lacking myc-UBQLN1 (Fig 1C). Although myc-UBQLN1 did not co-immunoprecipitate Rv1926c-V5, Rv1926c-V5 did co-immunoprecipitate UBQLN1 when using an antibody recognizing V5, whereas minimal myc-UBQLN1 was detected when an isotype control antibody was used (Fig 1D). The amount of UBQLN1 that co-immunoprecipitate with Rv1926 was 27% of that which could be precipitated directly using the myc antibody. In the case of Rv1566-V5 and myc-UBQLN1, we could co-immunoprecipitate the proteins in both directions (Fig 1E), and in both cases 7% of the amount directly precipitated was co-immunoprecipitated. Thus, all of the MUPs tested co-immunoprecipitated in at least one direction with UBQLN1.

### UBQLN1 associates with Mtb *in vitro*

**Fig 2:**
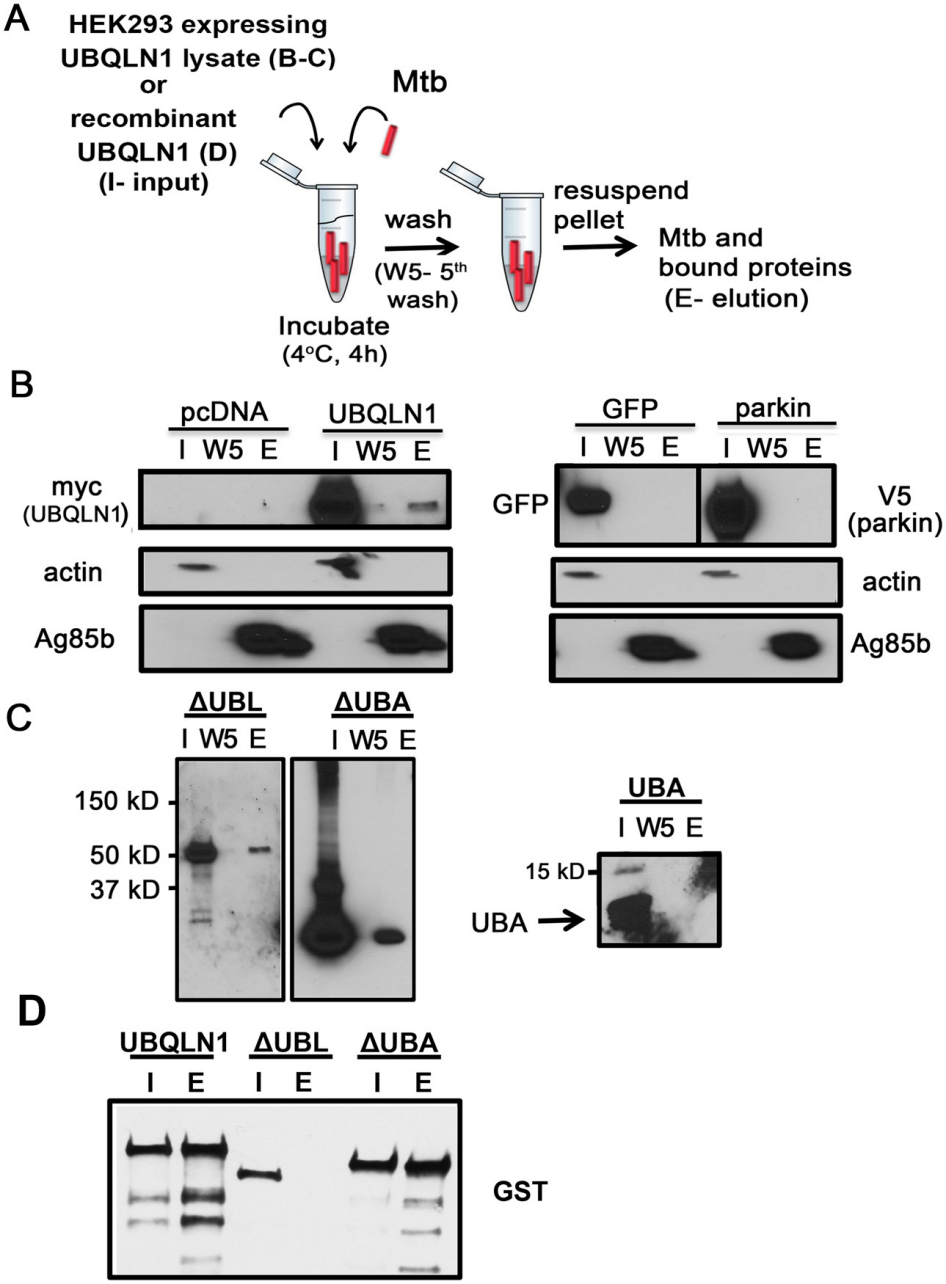
UBQLN1 binds Mtb *in vitro*. (A) Mtb-UBQLN1 binding assay. Mtb were incubated with HEK293 cell lysate (B, C) or recombinant protein (D) (input) and washed 5 times to remove unbound proteins. The pellet contains Mtb and associated host proteins (elution). (B) Mtb were incubated with lysate from HEK293 cells transfected with vector control, myc-UBQLN1, GFP, or V5-parkin. WB analysis of the input (I), 5^th^ wash (W5), and elution (E), probed with indicated antibodies. Actin is a loading control for the cell lysate and Ag85b for the bacterial pellet. (C) Mtb were incubated with lysate from HEK293 cells transfected with myc-UBQLN1-ΔUBA, myc-UBQLN1-ΔUBL, and myc-UBQLN1-UBA. WB containing the input, 5^th^ wash, and elution were incubated with anti-myc antibody. The predicted sizes of myc-UBQLN1-ΔUBL, myc-UBQLN1-ΔUBA, and myc-UBQLN1-UBA are 55 kD, 50 kD, and 6 kD, respectively. (D) Mtb were incubated with recombinant GST-UBQLN1, GST-UBQLN1-ΔUBL, or GST-UBQLN1-ΔUBA. WB containing the input and elution was probed with an anti-GST antibody.

Since UBQLN1 can interact with Mtb surface proteins, we reasoned that it might associate with Mtb in a cell free system. We incubated Mtb with cytosol from HEK293 cells that had been transfected with plasmid encoding UBQLN1, truncated versions of UBQLN1, or vector control (Fig 2A, 2B and 2C). After four hours of incubation, we washed the bacteria to remove unbound host proteins. Actin was removed after the first wash, whereas UBQLN1 remained associated with Mtb after five washes. As additional controls for specificity, we tested cytosol from HEK293 cells transfected with GFP or the E3 ligase parkin. In contrast to UBQLN1, neither GFP nor parkin associated with Mtb (Fig 2B). To test if Ubqln1 binds Mtb directly, we purified GST-UBLQN1 and added it to Mtb *in vitro*. As we found with HEK293 cell lysate, UBQLN1 remained associated with Mtb after five washes (Fig 2D).

To further understand how UBQLN1 interacts with Mtb, we examined the roles of the UBL and UBA domains. A recombinant, truncated version of UBQLN1 lacking the UBL domain (UBQLN1-ΔUBL) failed to bind Mtb, whereas the UBA domain mutant had preserved binding (Fig 2C). Although recombinant UBQLN1-ΔUBL did not bind Mtb *in vitro*, when UBQLN1-ΔUBL was expressed in HEK293 cells or yeast, it interacted with intact Mtb as well as MUPs (Fig 2D and S3 Fig). This suggests that in co-immunoprecipitation and Y2H experiments, additional proteins, including UBQLN family members, may bridge or stabilize the UBQLN1-Mtb and MUP interactions. When we expressed UBQLN1 lacking the UBA domain (UBQLN1-ΔUBA) in HEK293 cells, there were numerous products both smaller and larger than the predicted protein, suggesting that UBQLN1- ΔUBA was degraded and post-translationally modified. Degradation products associated with Mtb in the cell free system (Fig 2C) and also co-immunoprecipitated with MUPs (Rv1566 and Rv2911; S3 Fig). In the Y2H, UBQLN1-ΔUBA failed to interact with MUPs, perhaps related to its propensity for degradation (S3 Fig). The UBA domain alone failed to associate with Mtb (Fig 2C). In conclusion, Mtb associates with recombinant UBQLN1 *in vitro* and in a cell free system. The UBL domain appears important *in vitro*, although in the context of cytoplasm, it is dispensable, perhaps related to the ability of endogenous UBQLN family members or other adaptor proteins to multimerize and recruit the truncated protein. Determining the contribution of the UBA domain is confounded by the propensity for the truncated protein to be degraded, but the combined data suggest the UBA domain is not required.

### UBQLN1 associates with Mtb, restricts its growth, and promotes T cell activation in IFN-γ activated macrophages

**Fig 3:**
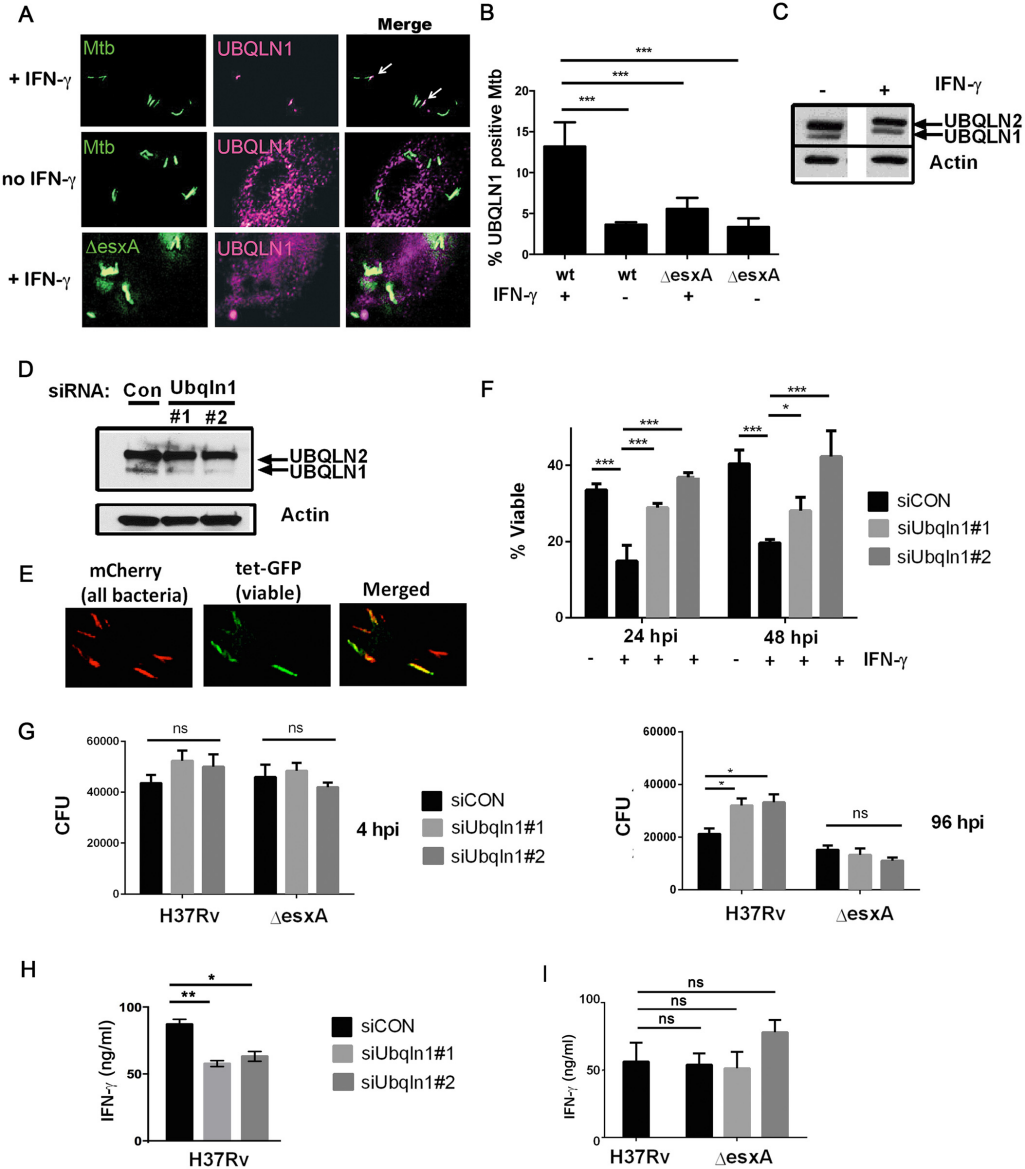
UBQLN1 localizes to Mtb, restricts its growth, and promotes the ability of macrophages to active CD4+ T cell. (A) BMDMs were infected with GFP-expressing wt Mtb or Δ*esxA* for 24h and immunostained with UBQLN1 antibody. IFN-**γ** was added 1d prior to infection as indicated. (B) Quantification of results from *A* from 3 independent experiments with at least 100 bacteria analyzed per experiment; ***P< 0.001, Fisher’s exact test, comparing the proportion of UBQLN1 positive versus negative Mtb in indicated samples. (C) Cellular lysate from BMDMs that were treated with IFN-**γ** as indicated were analyzed by WB with an antibody that recognizes UBQLN1 and UBQLN2. (D) BMDMs were transfected with siRNAs targeting UBQLN1 or a non-targeting control (siCON) and lysates were analyzed by WB using an antibody that recognizes UBQLN1 and UBQLN2. (C-D) Actin served as a loading control. (E) BMDMs were infected with Mtb live/dead strain and treated with AnTc 24h prior to fixation. (F) BMDMs were transfected with siRNAs targeting UBQLN1 or siCON and infected with live/dead Mtb strain. IFN-**γ** was added 1d prior to infection if indicated. %Viable bacteria were quantified 24 and 48 hpi. Data are from 2 independent experiments. *P<0.05; ***P<0.001, Fisher’s exact test, comparing the proportion of viable and non-viable bacteria in indicated samples. (G) BMDMs were treated with the indicated siRNAs three days prior to infection and then activated with IFN-**γ** one day prior to infection with H37Rv or Δ*esxA*. CFU were enumerated 4 and 96 hpi. *P< 0.05, unpaired Student’s *t*-test. Data are representative of three independent experiments. (H) BMDMs transfected with siRNA targeting UBQLN1 or siCON were infected with wt Mtb (H37Rv) (I) UBQLN1-silenced BMDMs and controls were infected with H37Rv or Δ*esxA*. (H-I) 24 hpi infected BMDMs were co-cultured with P25TCR-Tg CD4+ T cells, and IFN-**γ** was measured by ELISA. Data is representative of 3 independent experiments. *P<0.05, **P<0.005, unpaired Student’s *t*-test. (B, F, Γ, H) Results are mean +/- SEM; ns, not significant.

UBQLN1 interacted with Mtb proteins and was recruited to the bacteria from host cytosol. Therefore, it should co-localize with the bacteria during an infection. We used a UBQLN1-specific antibody to examine its localization by immunofluorescence microscopy. UBQLN1 was predominantly found in small cytoplasmic punctae (S4 Fig). When we examined the relationship of UBQLN1 to Mtb in unactivated macrophages, we found a low level of co-localization. The association was enhanced in IFN-**γ** activated macrophages, in which we found UBQLN1 co-localized with 13% of Mtb (Fig 3A and 3B). Although the association of UBQLN1 with Mtb was more prominent in IFN-**γ** activated macrophages, the protein was present at equivalent levels in untreated macrophages (Fig 3C) and the overall cellular distribution of UBQLN1 looked similar in activated and naïve cells (S4 Fig). In IFN-**γ** activated macrophages, there was little co-localization of UBQLN1 with an Mtb Δ*esxA* mutant, which does not permeabilize the phagosome (Fig 3B) [12,14], suggesting that the association of UBQLN1 with Mtb requires phagosomal damage to provide access to the bacteria.

To determine whether UBQLN1 plays a role during infection, we examined macrophages in which UBQLN1 was depleted using RNAi. We used two different siRNAs which reduced UBQLN1 levels by 88% (siRNA #1) and 90% (siRNA #2; Fig 3D) based upon quantification using ImageJ and normalizing to actin levels. There was no significant effect on UBQLN2 levels. Following UBQLN1-depletion, bone marrow-derived macrophages (BMDMs) were infected with a live/dead Mtb reporter strain that expresses mCherry constitutively and GFP under control of a tetracycline-inducible promoter [29]. After treatment with anhydrotetracycline (AnTc), metabolically active bacteria express both GFP and mCherry, whereas dead bacteria only express mCherry (Fig 3E). As expected, control macrophages that were activated with IFN-**γ** restrained growth of Mtb. In contrast, UBQLN1-depleted macrophages were impaired in restricting Mtb growth (Fig 3F). We corroborated these results by plating for colony forming units (CFU). UBQLN1 silencing had no effect on bacterial uptake 4 hours post infection (hpi), but it rendered IFN-**γ** activated macrophages defective in their ability to control Mtb at 96 hpi (Fig 3G). UBQLN1 was also required to control Mtb in IFN-**γ** activated RAW264.7 (RAW) cells, a murine macrophage cell line (S5 Fig). In accordance with the localization data, there was no effect of UBQLN1 silencing in unactivated macrophages (S5 Fig) or on infections with the Δ*esxA* mutant (Fig 3G and S5 Fig). To determine whether UBQLN1 controls the general antimicrobial capacity of macrophages, we examined its effect on the intracellular growth of *Staphylococcus aureus* and *Mycobacterium smegmatis*. There was no effect of depleting UBQLN1 on *S*. *aureus* or *M*. *smegmatis* survival (S6 Fig). Thus, although UBQLN1 restricts growth of Mtb, it is not universally required for the antimicrobial capacity of macrophages towards all bacteria.

Because MUPs also bound UBQLN2 and UBQLN4, we examined whether these family members play a role in controlling Mtb replication in macrophages. We found that UBQLN2, but not UBQLN4, was present in macrophage lysate (S4 Fig). We attempted to silence UBQLN2 using siRNA, but we only achieved limited depletion, which did not result in any change in mycobacterial CFU (S4 Fig). Thus, UBQLN1 restricts Mtb growth in activated macrophages, and we were unable to draw definitive conclusions about UBQLN2.

Another important function of macrophages is to present antigen presentation to and activate T cells. We examined the ability of UBQLN1-silenced macrophages to activate Th1 polarized CD4+ T cells using P25TCR-Tg T cells which recognize the peptide 25 epitope of Mtb Antigen 85B. T cell production of IFN-**γ** was antigen specific, as IFN-**γ** was not detected when macrophages were infected with a strain lacking Antigen 85b (Δ*fbpB*). Notably, IFN-**γ** secretion was lower when P25TCR-Tg T cells were co-cultured with UBQLN1-depleted macrophages compared to co-culture with controls (Fig 3H). There was no effect of UBQLN1 silencing on MHCII surface expression (S7 Fig). UBQLN1 silencing did not impair the ability of macrophages infected with Δ*esxA* to activate T cells (Fig 3I), consistent with the idea that UBQLN1 acts on bacilli that access the cytosol. This also argues that impaired T cell activation is not caused by a non-specific effect such as decreased macrophage viability. In summary, UBQLN1 limits Mtb replication and enhances the ability of macrophages to activate effector T cells.

### UBQLN1 co-localizes with Mtb that are cleared via autophagy

**Fig 4:**
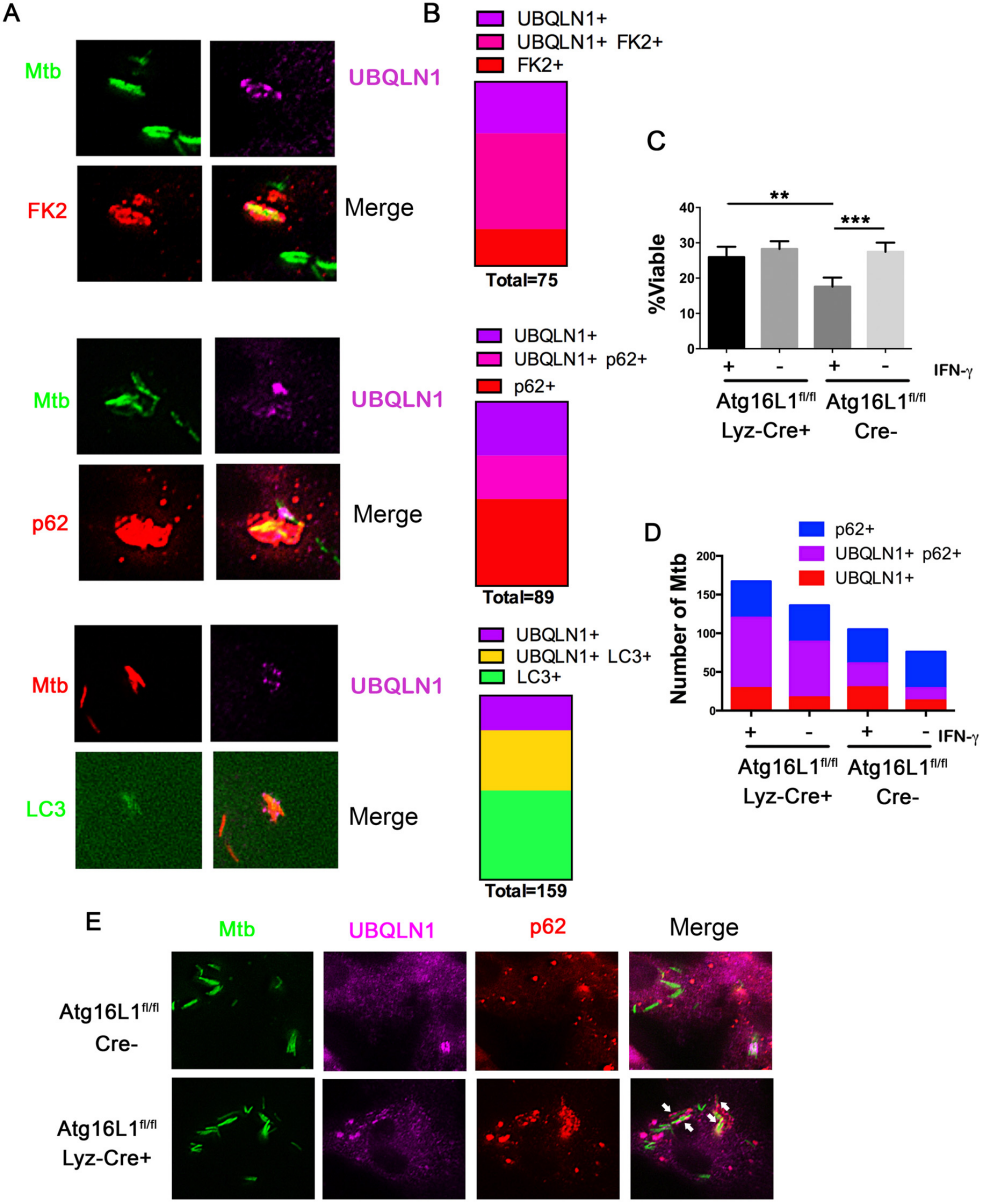
UBQLN1 co-localizes with Mtb that are cleared through autophagy. (A) BMDMs treated with IFN-**γ** and infected for 24h with GFP-Mtb were immunostained for UBQLN1 and either ubiquitin (using the FK2 antibody) or p62. GFP-LC3 BMDMs infected with DsRed-Mtb were immunostained for UBQLN1. (B) Quantification of those Mtb that co-localize with at least one of the cellular markers shown in A. More than 375 total bacteria were counted in two independent experiments, and the number co-localizing with at least one marker is indicated as “total” under each bar. (C) Wt macrophages (Atg16L1^flox/flox^; Cre-) and autophagy-deficient macrophages (Atg16L1^flox/flox^ Lyz-cre; Cre+) were activated with IFN-**γ** or not, followed by infection with the live/dead Mtb reporter strain. % viable was quantified after addition of AnTc. **P<0.005, ***P<0.0001, Fisher’s exact test (in which the proportions of viable to non-viable Mtb was compared). (D) Atg16L1^flox/flox^ Cre+ and Cre- BMDMs treated with IFN-**γ** and infected for 24h with GFP-Mtb were immunostained for UBQLN1 and p62. The number of Mtb co-localizating with the cellular markers is shown. (C-D) At least 100 bacteria were analyzed in three independent experiments. (E) Images from IFN-**γ** treated BMDMs of indicated genotype infected with GFP-Mtb and immunostained with UBQLN1 and p62. Arrows indicates p62+ UBQLN1+ Mtb.

The above findings demonstrate that UBQLN1 recognition of Mtb and its role in controlling Mtb replication depends upon the bacterial ESX-1 system and host IFN-**γ** activation, both of which promote Mtb xenophagy [4,14]. We examined the accumulation of LC3-II by western blotting in uninfected macrophages, and found that UBQLN1 was required for basal autophagy in uninfected, IFN-**γ** activated BMDMs (S5 Fig). To evaluate whether UBQLN1 is involved in xenophagy, we examined the localization of UBQLN1 positive Mtb relative to autophagy components (Fig 4A). In activated macrophages, we found that more than 60% of the UBQLN1 positive Mtb co-localized with ubiquitinated proteins, which surround mycobacteria that damage or escape the phagosome [8,12,30]. 45% of the UBQLN1 positive bacteria also co-localized with p62, an autophagy adaptor that is recruited to ubiquitinated bacteria and binds LC3. Finally, more than 60% of the UBQLN1 positive bacteria co-localized with LC3. Likewise, a prominent fraction of the bacteria that associated with FK2, p62, or LC3 were UBQLN1 positive (Fig 4B), all of which is consistent with the idea that UBQLN1 functions in autophagy-mediated clearance of Mtb.

We next asked whether UBQLN1 positive Mtb are trafficked through the autophagy pathway. Initially, we examined whether IFN-**γ**-mediated control of Mtb depends upon Atg16L1, a component of the autophagy elongation complex that conjugates LC3 to phosphatidylethanolamine on the incipient autophagosome. IFN-**γ** restricted bacterial growth in wild type (wt) macrophages (Atg16L1^flox/flox^ Cre-), but not in autophagy-deficient macrophages (Atg16L1^flox/flox^ Lyz-Cre^+^) (Fig 4C), demonstrating that autophagy is required for the antibacterial properties of IFN-**γ**. In IFN-**γ** activated macrophages, there were more p62-positive Mtb in autophagy-deficient cells than controls (22.8 +/- 2.2% versus 11.8 +/- 5.3%; p<0.03), consistent with the idea that p62 associates with Mtb that are directed to the autolysosome where p62 is subsequently degraded. Similarly, IFN-**γ**-activated, Atg16L1-deficient macrophages contained more UBQLN1 positive Mtb compared to autophagy-competent cells (20.4 +/- 4.7% vs 10.5 +/- 2.7%, p<0.04), suggesting that UBQLN1-decorated Mtb are also targeted for xenophagy. Moreover, the fraction of UBQLN1 positive bacteria that were also positive for p62 increased from ~50% in wt macrophages to ~80% in the autophagy-deficient cells (Fig 4D and 4E), implying that double positive bacteria are eliminated by autophagy. Combined, these data support the idea that UBQLN1 associates with bacteria that are cleared through autophagy.

### UBQLN1 promotes Mtb ubiquitination independently of parkin

**Fig 5:**
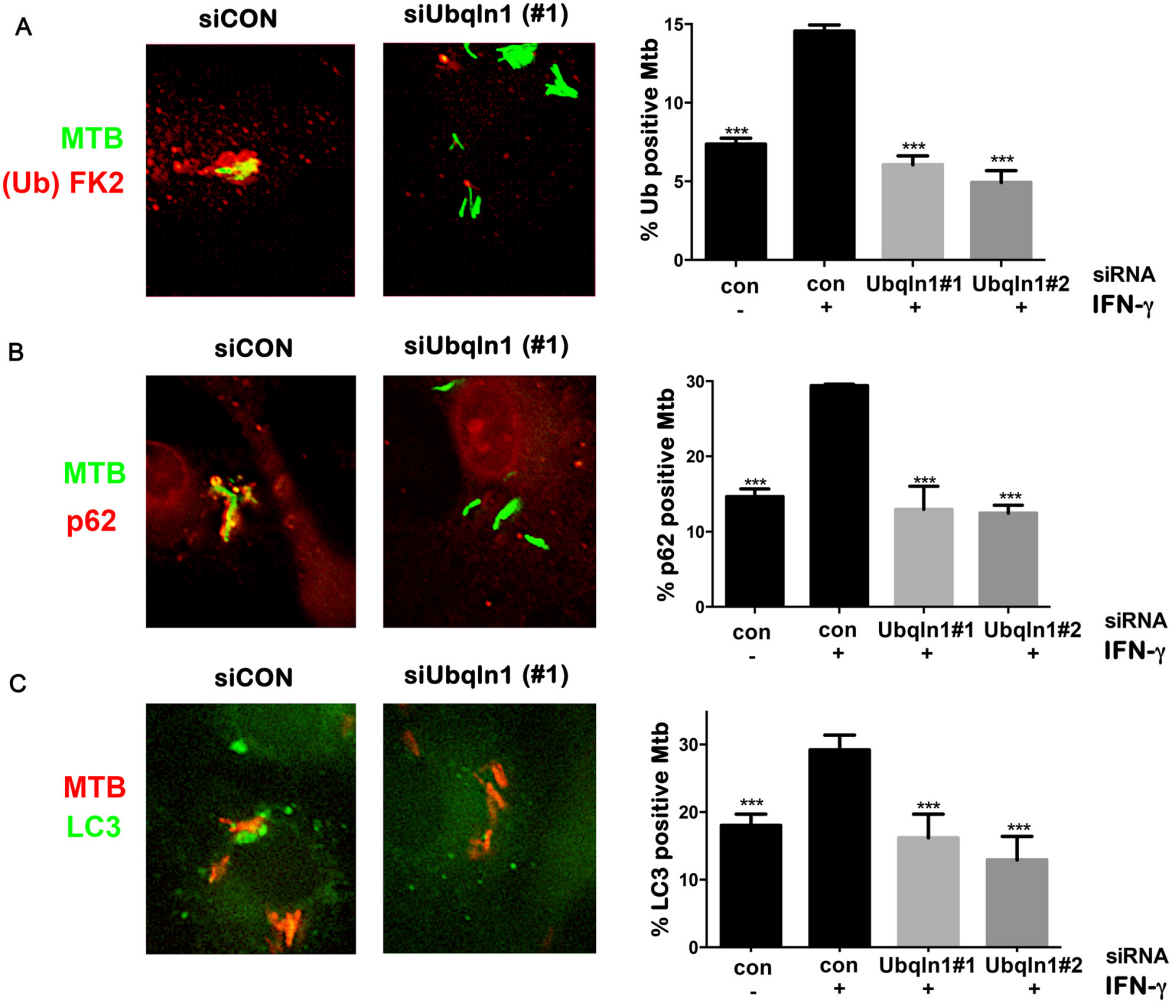
UBQLN1 promotes ubiquitin, p62, and LC3 recruitment to Mtb. (A-C) BMDMs transfected with siRNA targeting UBQLN1 or control siRNA. Images show IFN-**γ** treated macrophages infected with GFP-Mtb (A-B) or DsRed-Mtb (C) for 24h. Cells were immunostained for ubiquitin (Ub) with the FK2 antibody (A) or p62 (B), which are shown in red. (C) GFP-LC3 BMDMs were infected with DsRed-Mtb. LC3 is shown in green, whereas Mtb are red. (A-C) Quantification shows the percentage of Mtb that colocalized with the indicated cellular marker in macrophages that were treated with IFN-**γ** as indicated. Results are mean +/- SEM from two independent experiments, with at least 100 bacteria analyzed per experiment. ***P<0.001, Fisher’s exact test (in which the proportion of Mtb associated with the cellular marker in a given condition was compared to the proportion found in the sample treated with siCON and IFN-**γ**).

UBQLN1 binds mono and polyubiquitin [31], so we thought that it might be recruited to ubiquitinated Mtb, like the autophagy adaptors p62 and human NDP52. Alternatively, since UBQLN1 can bind MUPs, we also thought that UBQLN1 might directly recognize Mtb and act upstream of ubiquitination. To distinguish these possibilities, we examined ubiquitination of Mtb in UBQLN1-silenced BMDMs. As expected, IFN-**γ** promoted the ubiquitination of Mtb. Notably, the recruitment of ubiquitin to Mtb in activated cells depended upon UBQLN1. Similarly, the enhanced co-localization of p62 and LC3 with Mtb seen in IFN-**γ** treated macrophages was blunted in cells lacking UBQLN1 (Fig 5A, 5B and 5C). Thus, we conclude that UBQLN1 promotes Mtb-associated ubiquitination and subsequent recruitment of adaptor proteins and the autophagy machinery during IFN-**γ** promoted xenophagy.

**Fig 6:**
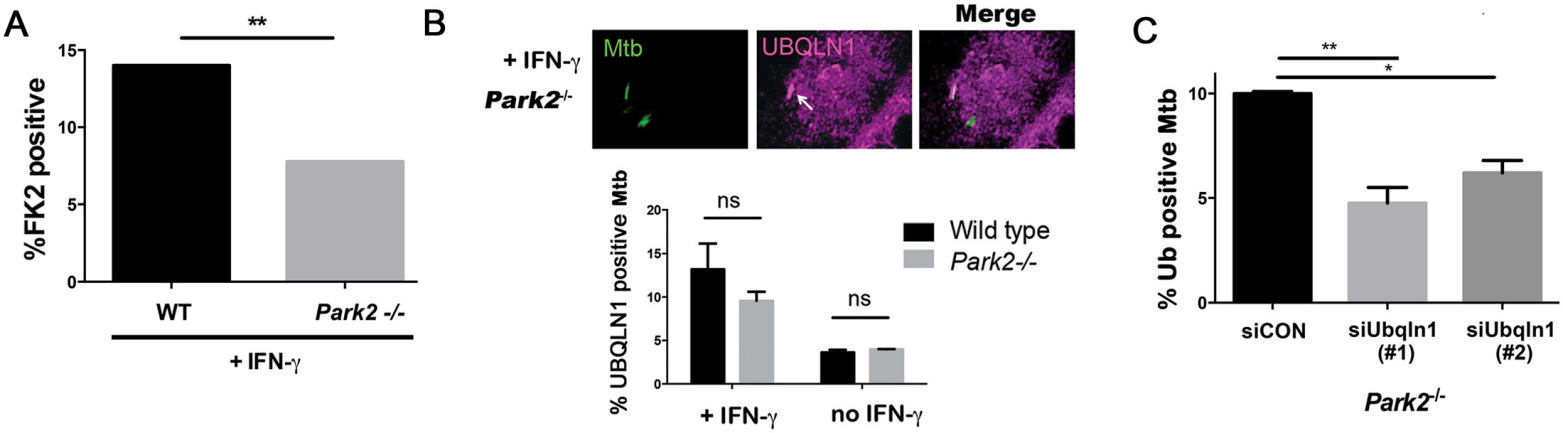
UBQLN1 acts in parallel to parkin. (A) WT and *Park2* ^-/-^ BMDMs were pretreated with IFN-**γ**, infected with GFP-Mtb for 24h, and immunostained with the FK2 antibody. Bacterial colocalization with FK2 immunoreactivity was quantified from 2 independent experiments. (B) *Park2* ^*-/-*^ BMDMs pretreated or not treated with IFN-**γ** were infected with GFP-Mtb for 24h and immunostained with UBQLN1 specific antibody; Arrows indicate Mtb co-localized with UBQLN1. ns- not significant. (C) *Park2* ^*-/-*^ BMDMs were transfected with siRNA targeting UBQLN1 or control siRNA 3d prior to infection, treated with IFN-**γ,** and infected with GFP-Mtb for 24h prior to immunostaining for ubiquitin (FK2 antibody). Ubiquitin (FK2+) bacteria were quantified from two independent experiments. (A, C) **P<0.01, Fisher’s exact test (comparing the proportion of FK2 positive bacteria in indicated samples). Results are mean +/- SEM. A minimum of 100 Mtb were counted in each experiment.

The E3 ligase parkin is required for ubiquitin recruitment to Mtb in naïve macrophages [17], and we found that it also played a role in IFN-**γ** activated macrophages. In activated macrophages, the parkin knockout macrophages had half as many ubiquitin positive Mtb as wt macrophages (Fig 6A). We asked whether parkin is also required for UBQLN1 recruitment to Mtb. While there was a slight trend towards decreased UBQLN1 positive Mtb in the parkin mutant, it was not statistically significant (Fig 6B). This suggests that UBQLN1 largely localizes independently of parkin and ubiquitination. In addition, in macrophages lacking parkin we found that UBQLN1 silencing diminished ubiquitin association with Mtb, much as it did in wt macrophages (Fig 6C). Combined, these results suggest that in activated macrophages, UBQLN1 and parkin act independently to recruit ubiquitinated proteins to Mtb.

## Discussion

Although *in vitro* autophagy makes only a limited contribution to macrophage control of Mtb, *in vivo* mice are profoundly susceptible to Mtb infection if they lack parkin or autophagy proteins in myeloid cells [8,17,32]. In addition to controlling bacterial replication, xenophagy modulates cytokine responses and promotes MHCII antigen presentation [32,33]. Here, we provide multiple lines of evidence that UBQLN1 associates with Mtb and links them to the autophagy machinery: UBQLN1 binds MUPs, binds Mtb *in vitro*, and localizes to Mtb during infection. The association of UBQLN1 with Mtb depends upon EsxA, which is likely related to the role of EsxA in damaging the phagosomal membrane; however, it is also possible that EsxA plays some other role in recruiting UBQLN1 to the bacteria. Consistent with a role for UBQNL1 in recognizing Mtb, in the absence of UBQLN1 there is less ubiquitin, p62, and LC3 recruited to Mtb, which correlates with impaired control of bacterial replication and diminished CD4+ T cell activation. Overall, our data are consistent with a model in which UBQLN1 recognizes Mtb that become accessible to the cytosol upon phagosomal damage; UBQLN1 then assists in recruiting the autophagy machinery.

Why did we only detect a role for UBQLN1 in IFN-**γ** activated macrophages when the protein is also present in unactivated macrophages? UBQLN1 probably does not function in the IFN-**γ** signaling pathway, as there was no difference in surface MHCII, an IFN-**γ** induced gene, between control and UBQLN1-silenced BMDMs (S7 Fig). The apparent selectivity for activated macrophages may simply reflect that we see very low levels xenophagy in unactivated cells, as evidenced by little FK2, p62, and LC3 association with Mtb in naïve macrophages (Fig 5). It may be difficult to detect significant differences when we alter the trafficking of this minor bacterial population, particularly using RNAi, which generates hypomorphic effects rather than complete loss of function. In addition, autolysosomes may be less antimicrobial in naïve cells compared with activated macrophages. Other investigators have reported higher levels of ubiquitin and LC3 association in unactivated macrophages (for example see [8,17]), which might be due to strain or other experimental differences. When we activated autophagy chemically with rapamycin, it resulted in considerable toxicity in siRNA-transfected, Mtb-infected cells making it difficult to draw any conclusions about the role of UBQLN1 in this context. However, we suspect UBQLN1 would play a detectable role in naive macrophages if they had robust xenophagy.

It is surprising that UBQLN1 recognizes so many Mtb proteins that appear to have little sequence conservation. In addition, there are likely to be additional MUPs as our Y2H screen only examined 399 Mtb proteins [26]. The MUPs do not appear to share much in common (S1 Table), although one thing almost all of the MUPs have is a predicted signal peptide. However, not all proteins containing signal peptides interact with UBQLN1, since UBQLN1 did not interact with Ag85B or the other proteins with signal sequences in our original Y2H screen [26]. Domain mutants of the MUPs have so far not been revealing, as all fragments have failed to interact. One possibility is that MUPs are prone to misfolding and aggregation. UBQLN1 is proposed to have chaperone activity [34], which may involve the STI1 motifs. Our findings that UBQLN1 promotes ubiquitination and adaptor recruitment is consistent with it playing an early role in xenophagy; however, it is possible that we identified the MUP-UBQLN1 interaction fortuitously, and MUPs are not what is responsible for recruiting UBQLN1 to Mtb. To verify the UBQLN1-MUP interactions or identify the *bona fide* UBQLN1 interacting proteins during an infection is technically challenging and an area of ongoing effort.

How do we envision UBQLN1 might work to promote xenophagy? One possibility is that it recruits or activates an E3 ligase, which ubiquitinates bacterial or host phagosomal components. In several studies, UBQLN1 has been shown to promote ubiquitination of target proteins [35–37], although the E3 ligases were not identified. For Mtb xenophagy, parkin was a strong candidate, since it was recently shown to be required for ubiquitination around Mtb [17]. However, UBQLN1 localized to Mtb and promoted ubiquitination even in the absence of parkin (Fig 6). In addition, UBQLN1 and parkin did not interact in Y2H or co-immunoprecipitation experiments. Hence, a different E3 ligase likely acts downstream of UBQLN1 and parallel to parkin. Another possibility is that UBQLN1 is part of an amplification loop that fosters the association of ubiquitinated proteins with Mtb; UBQLN1 might localize to Mtb by virtue of binding MUPs, misfolded or aggregated proteins, or ubiquitinated proteins and then recruit additional ubiquitinated proteins. Whether UBQLN2 also plays a role in Mtb xenophagy warrants additional investigation. Although we did not detect a role for UBQLN2, this may have been due to insufficient silencing.

In conclusion, we show that UBQLN1 is required for autophagy-mediated clearance of Mtb in response to IFN-**γ**. UBQLN1 associates with Mtb and promotes recruitment of ubiquitinated proteins, autophagy adaptors, and the autophagy machinery. We speculate that UBQLN1 recognizes MUPs or other aggregation prone proteins generated by Mtb or present in the phagosome. In doing so, UBQNL1 promotes innate resistance to Mtb in the same way that it protects cells from cytotoxicity due to aggregation-prone cellular proteins, such as APP, TDP-43, and polyQ-expanded Huntington’s disease protein [24]. Thus, in addition to their role in Alzheimer’s disease, polymorphisms in *UBQLN1* may influence susceptibility to tuberculosis, analogous to the dual role of *PARK2* (which encodes parkin) in Parkinson’s and leprosy [17,38]. *UBQLN2* mutations, which confer risk of ALS [23], are also worthy of further investigation. Overexpression of UBQLN1 ameliorates damage in murine models of stroke and Huntington’s disease [39,40]. Therefore, therapeutics that promote the activity of ubiquilins might have efficacy in neurodegenerative disorders and tuberculosis.

## Materials and Methods

Details regarding Y2H, cell culture conditions, bacterial and mouse strains, flow cytometry, T cell activation assay, recombinant protein production, antibodies, siRNAs, and plasmids can be found in the S1 Text.

### Co-immunoprecipitation and western blotting (WB)

For WB, cellular lysates were prepared in phosphate buffered saline (PBS) with 1% NP-40 and Halt Protease Inhibitor Cocktail (Thermo Scientific). For immunoprecipitations (IPs), HEK293 cells were lysed in PBS with 0.1% NP-40 and passed 25 times through a 25 gauge needle. Lysates were incubated with Sepharose G agarose beads (GE Healthcare) pre-bound with anti-Ubqln1 (Abcam) or anti-myc antibody (Fig 1C). Alternatively, Sepharose G Dynabeads coated with anti-myc, anti-V5, or control IgG antibodies were used (Fig 1D and 1E). Bound proteins were analyzed by WB.

### Intracellular bacterial growth assays

RAW cells or BMDMs were transfected with siRNAs for 2d (RAW) or 3d (BMDM) prior to infection. 200 U/ml murine IFN-**γ** (Gibco) was added 24h before infection as indicated. For Mtb and *M*. *smegmatis*, macrophages were infected with a single cell suspension at an MOI of 3 with at least three replicates per experiment as previously described [26]. 4 hpi macrophages were extensively washed, lysed with 0.1% Triton X-100 at indicated time points, and serial dilutions were plated on 7H11. CFU were counted 15–21 days later for Mtb and 2–3 days later for *M*. *smegmatis*. For *S*. *aureus* infection, bacteria were opsonized with human serum for 1h prior to infection. Macrophages were infected at an MOI of 1, washed extensively 30 min post-infection, and lysed in 0.1% Triton-X-100 at indicated time points. *S*. *aureus* were plated on Tryptic Soy Agar, and CFU were quantified the following day.

### Microscopic analysis of infected cells

RAW cells or BMDMs from C57BL/6, parkin KO, or LC3-GFP-expressing mice were plated in 8 well chamber slides. They were infected with a single cell suspension of Mtb expressing GFP, DsRed, or the live/dead plasmid at MOI of 5 followed by washing 4 hpi. At indicated time points, they were fixed in 1% paraformaldehyde (PFA)/PBS overnight. For live/dead analysis, 200 nM anhydrotetracycline (AnTc) was added 20–24 hours prior to fixation. % viable Mtb was calculated using the live/dead strain as a ratio of GFP-bright, metabolically active bacteria to total mCherry-positive bacteria. For immunofluorescence microscopy, macrophages were permeabilized with 0.1% Tween-20 prior to immunostaining with primary and corresponding secondary antibody. Images were acquired using the Nikon Eclipse TiE/B fluorescent microscope at 60x magnification and deconvoluted as previously described [26]. At least 100 bacteria from a minimum of three independent fields were examined per experiment. For reproduced images, in some cases background was subtracted by selecting an ROI (region of interest) where there were no cells. For reproduced images, contrast was altered equally for a given single channel image for all samples in an experiment. For example, the signal corresponding to UBLQN1 was contrast adjusted equally for all panels in Fig 3A and likewise for all panels in Fig 4A. In Fig 5, the Ub, p62, and LC3 channels are adjusted equivalently for the siCON panel and the siUBQNL1 panel. Similarly, the UBQLN1 and p62 channels were equally adjusted for the Cre+ and Cre- samples.

### Bacterial binding assay

1 x 10^6^ HEK293 were plated, transfected the following day, and lysed 2 days later in PBS with 0.1% NP-40 (lysis buffer) by passage through a 25 gauge needle 25 times in the same manner as co-immunoprecipitation experiments. Mtb were grown to between O.D. = 0.5 and 1, washed twice with PBS, and one O.D. of bacteria was mixed with HEK293 lysate and incubated at 4°C for 4 h. Bacteria were then pelleted, resuspended in 1 mL lysis buffer, transferred to a new tube, and washed 5 times with 1 mL lysis buffer. The resulting bacterial pellet was suspended in 100 μl lysis buffer, transferred to a new tube with SDS loading buffer, boiled, and analyzed by WB.

### Ethics statement

This study was conducted in strict accordance with the recommendations in the Guide for the Care and Use of Laboratory Animals of the National Institutes of Health. The New York University School of Medicine Institutional Animal Care and Use Committee approved all work with mice (protocol #130707–02). Euthanasia was performed prior to bone marrow harvest in accordance with the 2013 AVMA Guidelines for the Euthanasia of Animals.

## Supporting Information

S1 Fig The MUP-Ubiquilin interaction is specific. (A) EsxA, EsxB, and Ag85b (Gal4-DNA binding domain fusions; DBs) do not interact with human UBQLN1 (Gal4-activation domain fusion; ADs) in yeast two-hybrid (Y2H) assay. Growth on 3AT demonstrates an interaction. Absence of growth on cyclohexamide (CHX) indicates lack of autoactivation from the DB constructs. (B) MUP-DB fusions were tested with UBQLN1 (murine) and NDP52 (human and murine) AD fusions for interactions in Y2H.
(PDF)

S2 Fig Distribution of MUPs. The distribution of MUPs in culture filtrate (CF), membrane protein fraction (MB), or whole cell lysate (WCL) was obtained from a published mass spectrometry study [28]. In that study, the membrane protein fraction was generated by a Triton-X114 phase-separation to isolate lipophilic proteins. In the study, the abundance of a given protein in each fraction was calculated as follows: emPAI = 10^PAI^-1, where PAI (protein abundance index) was generated by dividing the observed parent ions with the number of theoretical observable peptides. The concentration of a protein in a fraction (mol%). was calculated by its emPAI value divided by the sum of all emPIA in the sample and multiplied by 100.
(PDF)

S3 Fig Interaction of MUPs with Ubqln1 truncations. (A) MUP-DBs (Gal4-DNA binding domain fusions) interact with Gal-4 activation domain fusions of full length human UBQLN1 and a truncated construct lacking the UBL domain, but not a version lacking the UBA domain in the yeast two-hybrid (Y2H) assay. Growth on plates containing 3AT demonstrates an interaction. Absence of growth on cyclohexamide (CHX) indicates lack of autoactivation from the DB constructs. (B and C) Western blot of input lysate and immunoprecipitations (IP) from HEK293 cells expressing Rv1566-V5 (B) or Rv2911-V5 (C) and myc-UBQLN1-ΔUBA. IP was performed using antibodies directed against myc, V5, or isotype control (IgG).
(PDF)

S4 Fig Immunofluorescence microscopy of UBQLN1. (A) UBQLN1 was visualized in Mtb infected BMDMs that were either naïve or activated with IFN-**γ**. The bottom panel shows the staining when the UBQLN1 primary antibody is absent. 60X, deconvoluted images without any background subtraction; contrast was enhanced equally for all panels. (B) RAW cells treated with control siRNA (siCON) or siRNA targeting UBQLN1 were examined by fluorescence microscopy for UBQLN1.
(PDF)

S5 Fig UBQLN1 restricts Mtb growth in IFN-γ activated RAW cells and is required for basal autophagy in uninfected macrophages. (A) RAW cells were treated with four different siRNAs targeting UBQLN1 or control siRNA at 30 nM for 2d. UBQLN1 was detected from cell lysate with Western blotting using an antibody that recognizes UBQLN1 and UBQLN2. siRNAs used in subsequent experiments (#1 and #2) are indicated. (B) Naïve or IFN-**γ** activated RAW cells treated with UBQLN1 siRNAs or a non-targeting control were infected with Mtb for 72 hours, and colony forming units (CFU) were determined from five independent wells. (C) Naïve or IFN-**γ** activated RAW cells treated with UBQLN1 siRNAs or non-targeting control were infected with the Δ*esxA* mutant for 72 hours, and CFU were determined from five independent wells. (D) HEK293 cells were transfected with a plasmid encoding UBQLN4. Cellular lysate from transfected HEK293 cells and untransfected RAW cells were examined by Western blotting for the presence of UBQLN4. Actin served as a loading control in A and D. (E) RAW cells were treated with siRNA pools targeting UBQLN1, UBQLN2, or control siRNA. UBQLN1 and UBQLN2 were detected from cell lysate with Western blotting using an antibody that recognizes UBQLN1 and UBQLN2. Individual siRNAs tested targeting UBQLN2 also did not achieve significant silencing. (F) IFN-**γ** treated RAW cells were treated with siRNAs pools targeting UBQLN1, UBQLN2, or control siRNA, infected with H37Rv, and CFU were quantified 72 hpi from five independent wells. Actin served as a loading control in A and D. For B, C and F, results are mean +/- S.E.M; *P<0.05, unpaired Student’s *t*-test. ns- not significant. (G) BMDMs were treated with UBQLN1 siRNA or a non-targeting control for two days, then treated with IFN-**γ**, and the following day they were incubated with 10 nM bafilomycin A1 in DMSO or DMSO alone for 24h prior to cell harvest for western blotting. The band in the LC3 blot corresponds to LC3-II, which was stabilized by the addition of bafilomycin. The LC3-II/actin ration is shown. There is less LC3-II in UBQLN1-silenced cells, which also contained more p62 than controls (UN indicates that LC3-II was undetectable).
(PDF)

S6 Fig UBQLN1 does not restrict growth of *M*. *smegmatis* or *S*. *aureus*. (A) RAW cells were transfected with siCon, siUbqln1#1, siUbqln1#2, or siTsg101. Tsg101 silencing was used as a positive control based upon our previous work [26,41]. Macrophages were treated with IFN-**γ** where indicated 1d prior to infection with *M*. *smegmatis*. CFU were plated 48 hpi. Data are combined from three independent experiments. (B) RAW cells transfected with siCon, siUbqln1#1, siUbqln1#2, or siTsg101 (positive control) were treated with IFN-**γ** where indicated 1d prior to infection with *S*. *aureus*. CFU were plated 30 min and 6 hpi. CFU values reflect bacterial growth (CFU at (6 hpi/30 min post-infection), normalized to control from three independent experiments. *P<0.05, unpaired Student’s *t*-test. Results are mean +/- S.E.M.
(PDF)

S7 Fig MHCII surface expression is unaffected by UBQLN1 silencing. BMDMs transfected with siCon, siUbqln1#1, or siUbqln1#2 were treated with IFN-**γ** 1d prior to infection with Mtb. 24hpi BMDMs were fixed and stained using Alexa Fluor 488 anti-mouse MHC class II. Flow cytometry was performed using a FACSCalibur, and FlowJo software was used for data analysis.
(PDF)

S1 Table Characteristics of the mycobacterial-ubiquilin-interacting proteins. (PDF)

S1 Text Supplementary materials and methods. This file also contains the supplementary references.
(DOCX)
