# Supplementary figures and images for: Ubiquilin 1 Promotes IFN-γ-Induced Xenophagy of Mycobacterium tuberculosis
Source: PLoS Pathog. 2015 Jul 30;11(7):e1005076. doi: 10.1371/journal.ppat.1005076 (PMC4520715; doi:10.1371/journal.ppat.1005076)

S1 Figure

**A**

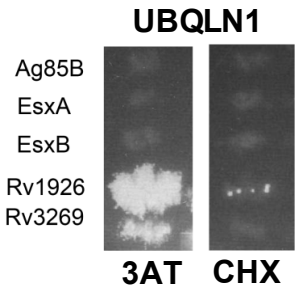

**B**

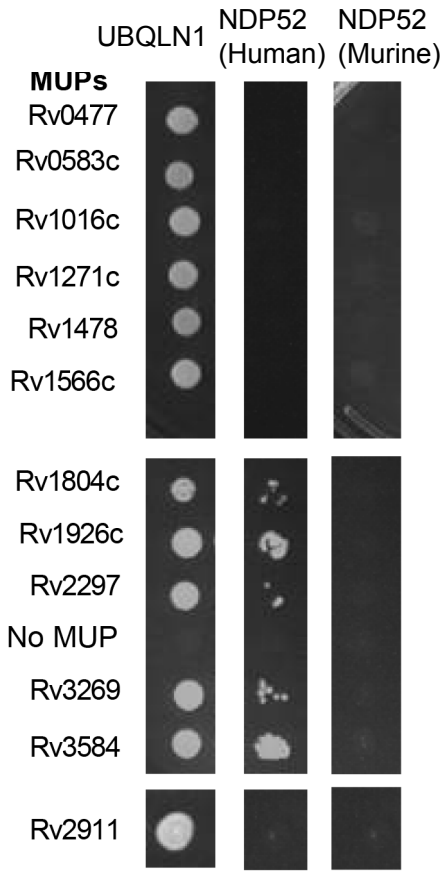

Supplement: S1 Fig — (A) EsxA, EsxB, and Ag85b (Gal4-DNA binding domain fusions; DBs) do not interact with human UBQLN1 (Gal4-activation domain fusion; ADs) in yeast two-hybrid (Y2H) assay. Growth on 3AT demonstrates an interaction. Absence of growth on cyclohexamide (CHX) indicates lack of autoactivation from the DB constructs. (B) MUP-DB fusions were tested with UBQLN1 (murine) and NDP52 (human and murine) AD fusions for interactions in Y2H. (PDF) [file ppat.1005076.s001.pdf]

S3 Figure

A

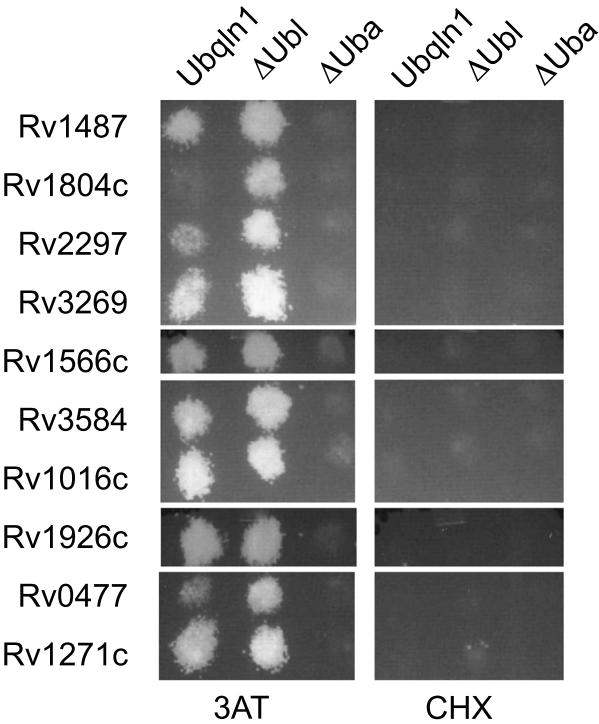

B

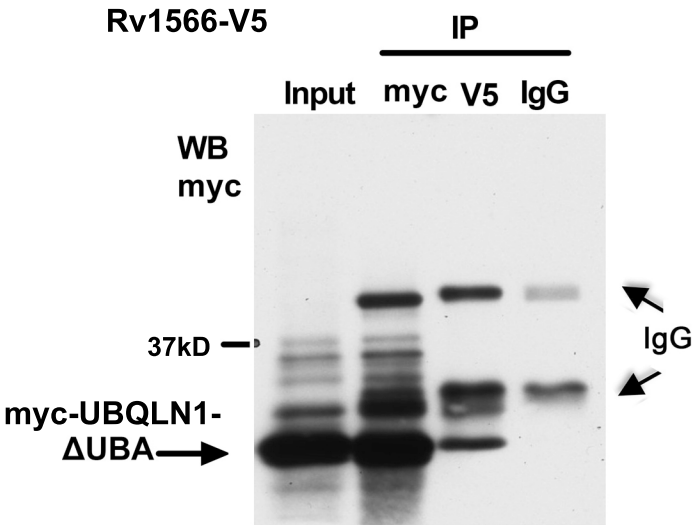

C

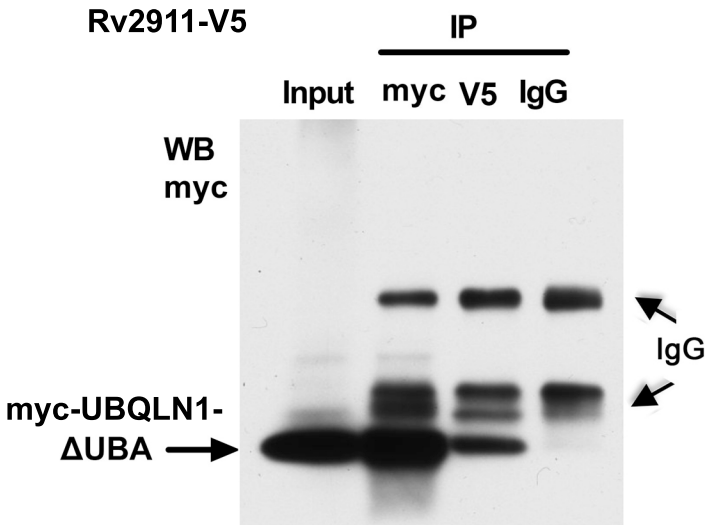

Supplement: S3 Fig — (A) MUP-DBs (Gal4-DNA binding domain fusions) interact with Gal-4 activation domain fusions of full length human UBQLN1 and a truncated construct lacking the UBL domain, but not a version lacking the UBA domain in the yeast two-hybrid (Y2H) assay. Growth on plates containing 3AT demonstrates an interaction. Absence of growth on cyclohexamide (CHX) indicates lack of autoactivation from the DB constructs. (B and C) Western blot of input lysate and immunoprecipitations (IP) from HEK293 cells expressing Rv1566-V5 (B) or Rv2911-V5 (C) and myc-UBQLN1-ΔUBA. IP was performed using antibodies directed against myc, V5, or isotype control (IgG). (PDF) [file ppat.1005076.s003.pdf]

**S4 Figure**

**A**

**UBQLN1  
(no IFN- $\gamma$ )**

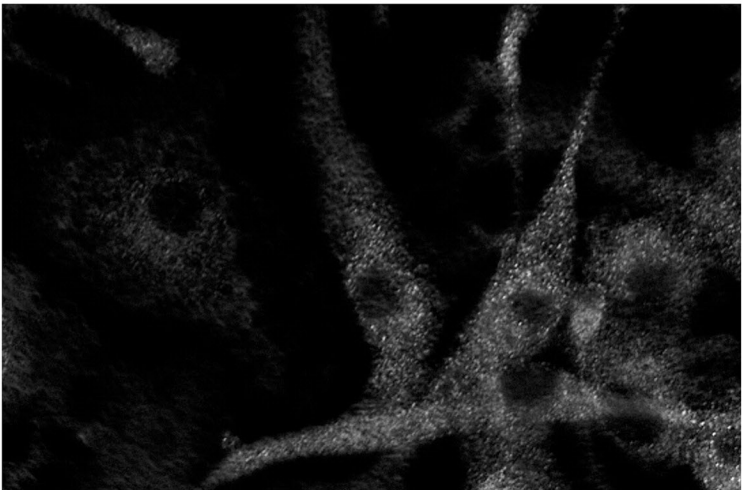

**UBQLN1  
(+ IFN- $\gamma$ )**

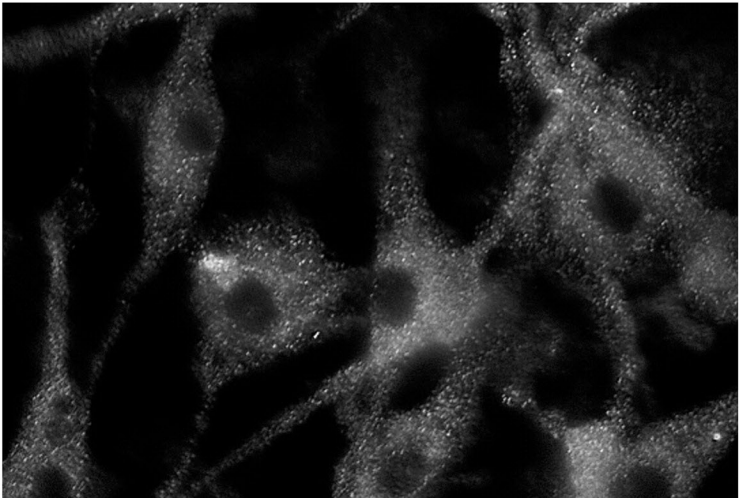

**No UBQLN1  
primary antibody**

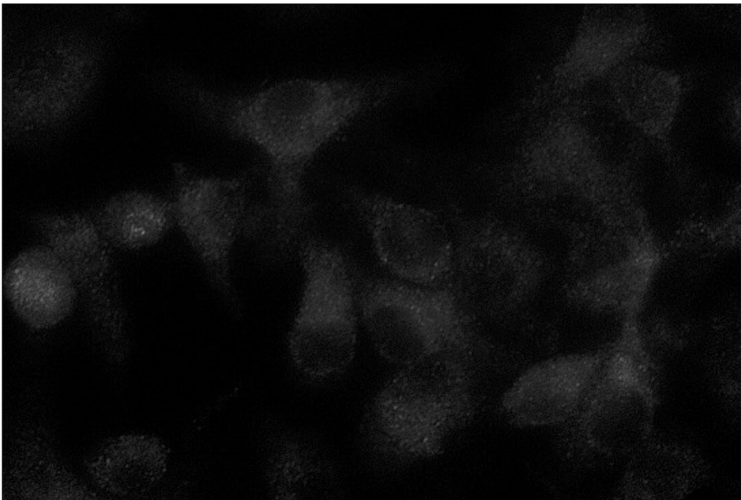

**B**

**siCON**

**siUBQLN1**

**UBQLN1**

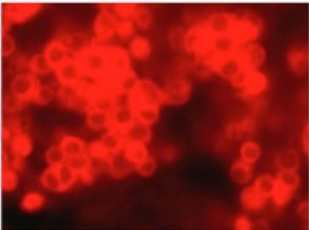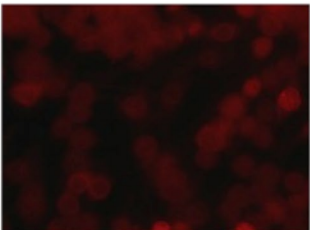

Supplement: S4 Fig — (A) UBQLN1 was visualized in Mtb infected BMDMs that were either naïve or activated with IFN-γ. The bottom panel shows the staining when the UBQLN1 primary antibody is absent. 60X, deconvoluted images without any background subtraction; contrast was enhanced equally for all panels. (B) RAW cells treated with control siRNA (siCON) or siRNA targeting UBQLN1 were examined by fluorescence microscopy for UBQLN1. (PDF) [file ppat.1005076.s004.pdf]

S5 Figure

A

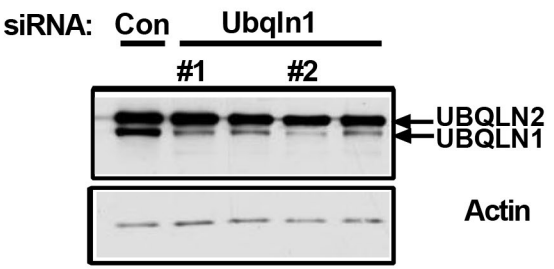

B

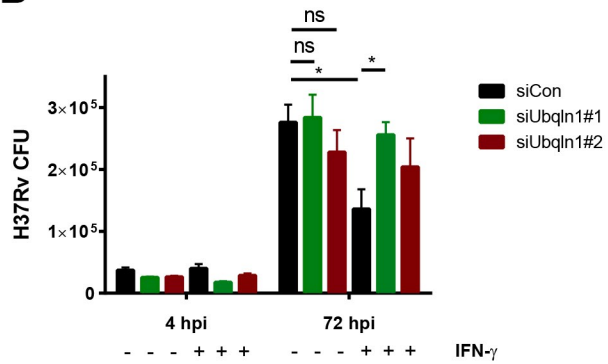

C

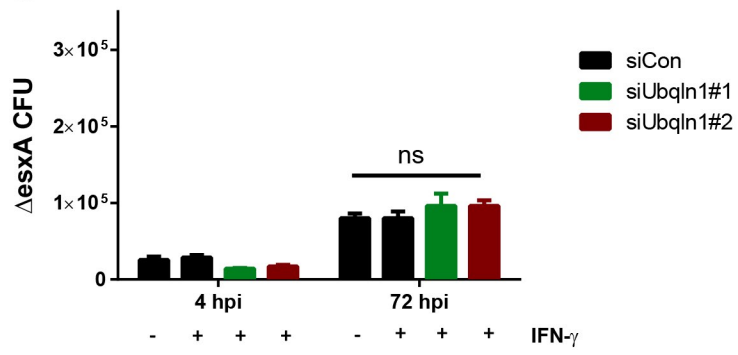

D

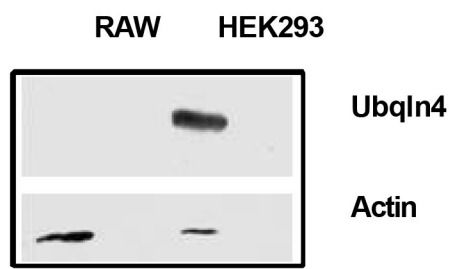

E

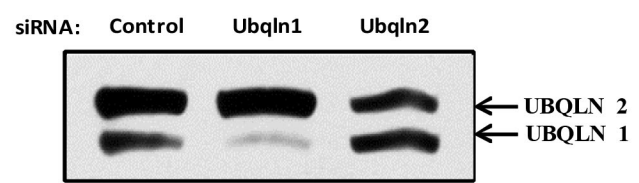

F

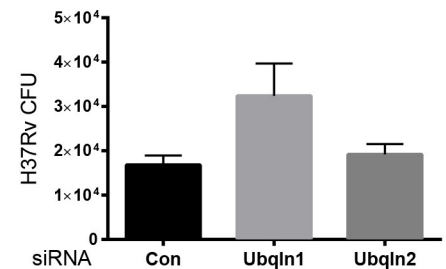

G

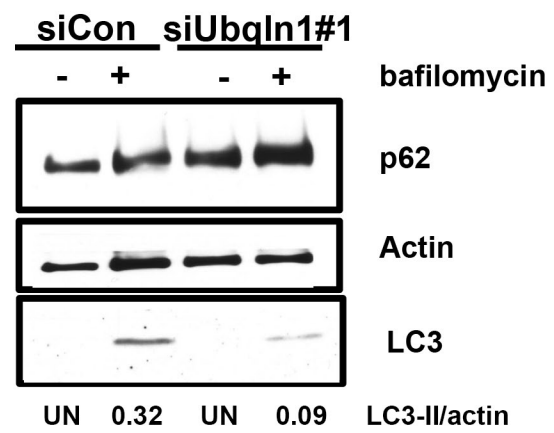

Supplement: S5 Fig — (A) RAW cells were treated with four different siRNAs targeting UBQLN1 or control siRNA at 30 nM for 2d. UBQLN1 was detected from cell lysate with Western blotting using an antibody that recognizes UBQLN1 and UBQLN2. siRNAs used in subsequent experiments (#1 and #2) are indicated. (B) Naïve or IFN-γ activated RAW cells treated with UBQLN1 siRNAs or a non-targeting control were infected with Mtb for 72 hours, and colony forming units (CFU) were determined from five independent wells. (C) Naïve or IFN-γ activated RAW cells treated with UBQLN1 siRNAs or non-targeting control were infected with the ΔesxA mutant for 72 hours, and CFU were determined from five independent wells. (D) HEK293 cells were transfected with a plasmid encoding UBQLN4. Cellular lysate from transfected HEK293 cells and untransfected RAW cells were examined by Western blotting for the presence of UBQLN4. Actin served as a loading control in A and D. (E) RAW cells were treated with siRNA pools targeting UBQLN1, UBQLN2, or control siRNA. UBQLN1 and UBQLN2 were detected from cell lysate with Western blotting using an antibody that recognizes UBQLN1 and UBQLN2. Individual siRNAs tested targeting UBQLN2 also did not achieve significant silencing. (F) IFN-γ treated RAW cells were treated with siRNAs pools targeting UBQLN1, UBQLN2, or control siRNA, infected with H37Rv, and CFU were quantified 72 hpi from five independent wells. Actin served as a loading control in A and D. For B, C and F, results are mean +/- S.E.M; *P<0.05, unpaired Student’s t-test. ns- not significant. (G) BMDMs were treated with UBQLN1 siRNA or a non-targeting control for two days, then treated with IFN-γ, and the following day they were incubated with 10 nM bafilomycin A1 in DMSO or DMSO alone for 24h prior to cell harvest for western blotting. The band in the LC3 blot corresponds to LC3-II, which was stabilized by the addition of bafilomycin. The LC3-II/actin ration is shown. There is less LC3-II in UBQLN1-silenced cells, which also [file ppat.1005076.s005.pdf]

# S6 Figure

A

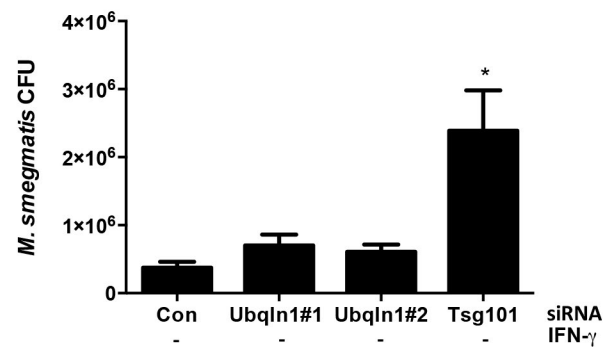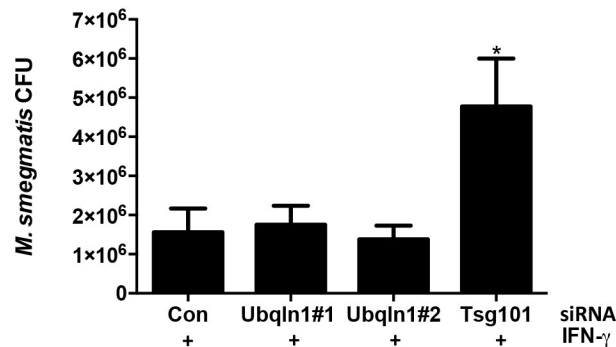

B

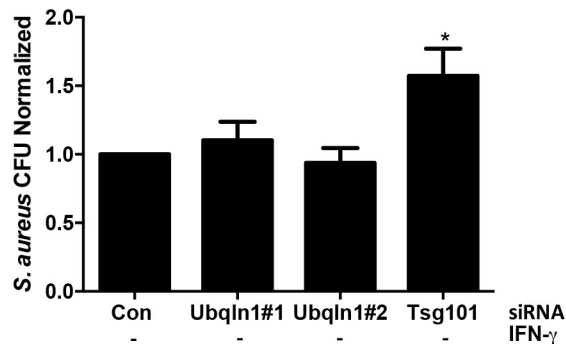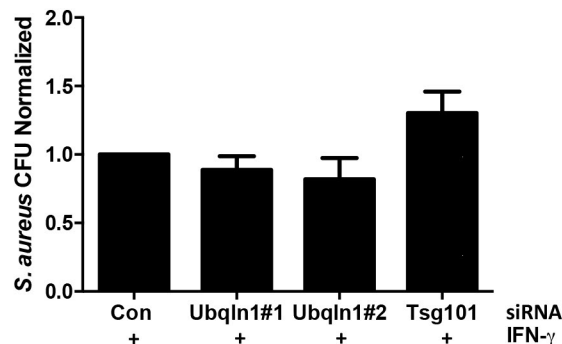

Supplement: S6 Fig — (A) RAW cells were transfected with siCon, siUbqln1#1, siUbqln1#2, or siTsg101. Tsg101 silencing was used as a positive control based upon our previous work [26,41]. Macrophages were treated with IFN-γ where indicated 1d prior to infection with M. smegmatis. CFU were plated 48 hpi. Data are combined from three independent experiments. (B) RAW cells transfected with siCon, siUbqln1#1, siUbqln1#2, or siTsg101 (positive control) were treated with IFN-γ where indicated 1d prior to infection with S. aureus. CFU were plated 30 min and 6 hpi. CFU values reflect bacterial growth (CFU at (6 hpi/30 min post-infection), normalized to control from three independent experiments. *P<0.05, unpaired Student’s t-test. Results are mean +/- S.E.M. (PDF) [file ppat.1005076.s006.pdf]

## S7 Figure

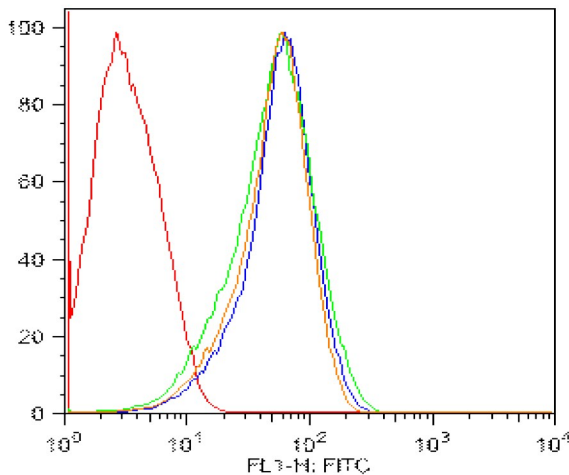

- 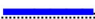 siCon
- 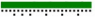 siUbqln1#1
- 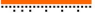 siUbqln1#2
- 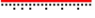 No stain control

Supplement: S7 Fig — BMDMs transfected with siCon, siUbqln1#1, or siUbqln1#2 were treated with IFN-γ 1d prior to infection with Mtb. 24hpi BMDMs were fixed and stained using Alexa Fluor 488 anti-mouse MHC class II. Flow cytometry was performed using a FACSCalibur, and FlowJo software was used for data analysis. (PDF) [file ppat.1005076.s007.pdf]
