# Supplementary material for: Ubiquilin 1 Promotes IFN-γ-Induced Xenophagy of Mycobacterium tuberculosis
Source: PLoS Pathog. 2015 Jul 30;11(7):e1005076. doi: 10.1371/journal.ppat.1005076 (PMC4520715; doi:10.1371/journal.ppat.1005076)

# S2 Figure

## Predominantly in Culture Filtrate (CF)

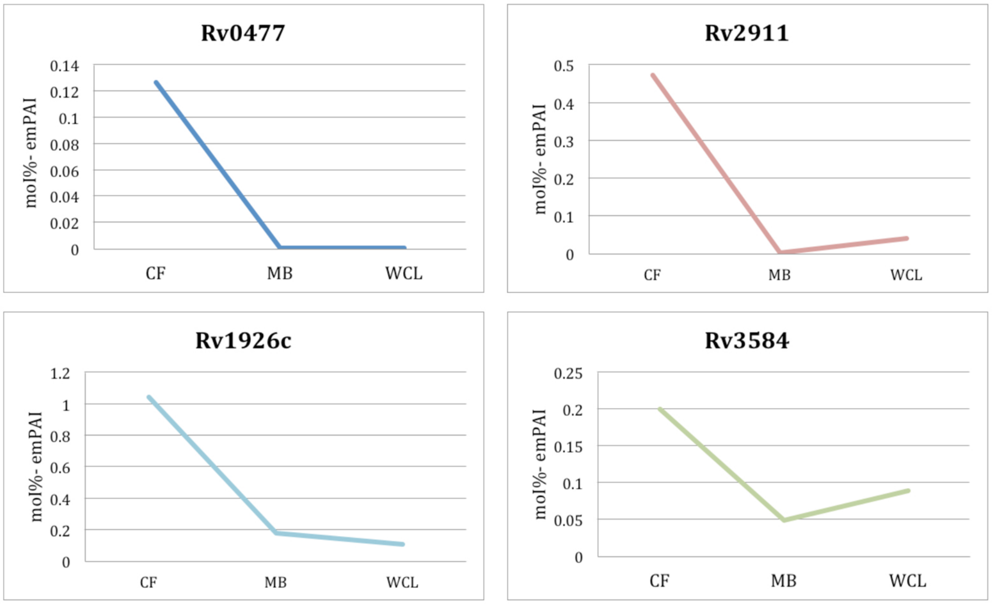


## Enriched in Membrane Fraction (MB)

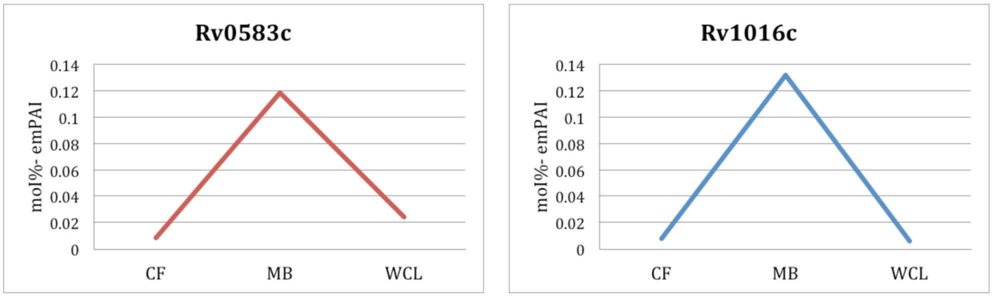


## Predominantly in Whole Cell Lysate (WCL)

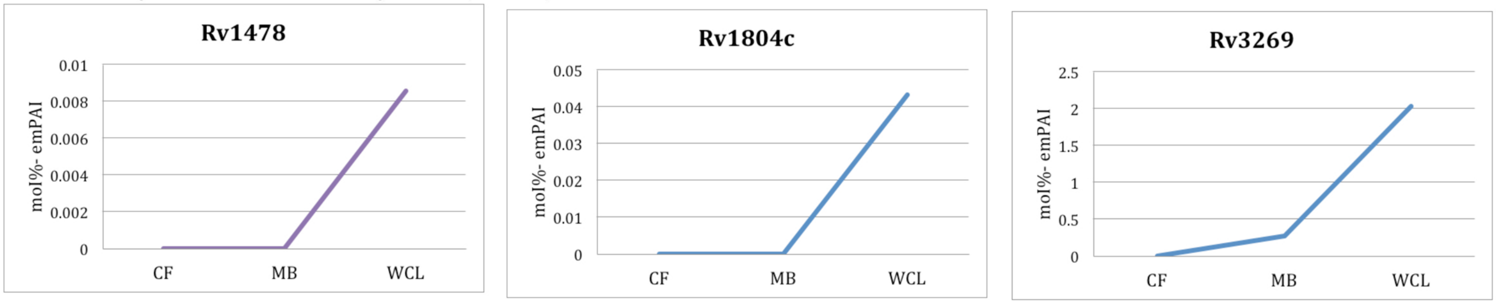


## MB and WCL

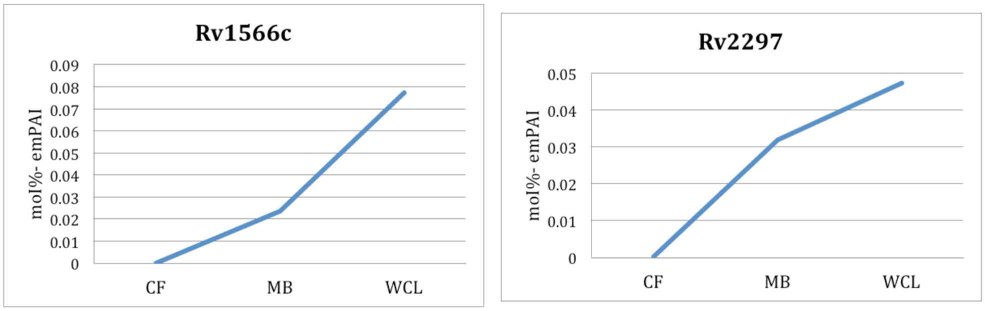

Supplement: S2 Fig — The distribution of MUPs in culture filtrate (CF), membrane protein fraction (MB), or whole cell lysate (WCL) was obtained from a published mass spectrometry study [28]. In that study, the membrane protein fraction was generated by a Triton-X114 phase-separation to isolate lipophilic proteins. In the study, the abundance of a given protein in each fraction was calculated as follows: emPAI = 10PAI-1, where PAI (protein abundance index) was generated by dividing the observed parent ions with the number of theoretical observable peptides. The concentration of a protein in a fraction (mol%). was calculated by its emPAI value divided by the sum of all emPIA in the sample and multiplied by 100. (PDF) [file ppat.1005076.s002.pdf]
