# Supplementary material for: Ubiquilin 1 Promotes IFN-γ-Induced Xenophagy of Mycobacterium tuberculosis
Source: PLoS Pathog. 2015 Jul 30;11(7):e1005076. doi: 10.1371/journal.ppat.1005076 (PMC4520715; doi:10.1371/journal.ppat.1005076)
Supplement: S1 Table — (PDF) [file ppat.1005076.s008.pdf]

S1 Table. Characteristics of the Mycobacterial-Ubiquilin-Interacting Proteins

Rv Number	Name	Function	Length (aa)	pI	ss	CF	Domains
Rv0477	UfaA1	Cyclopropane-fatty-acyl-phospholipid synthetase	148	4.4	Y	Y (10-12)	
Rv0583c	LpqN	Probable conserved lipoprotein	228	4.4	Y	Y (10, 11)	
Rv1016c	LpqT	Probable conserved lipoprotein	226	7.1	Y	Y (11)	
Rv1271c	Rv1271c	Conserved hypothetical secreted protein	113	4.6	Y	Y (12)	
Rv1478	RipB	Peptidoglycan endopeptidase	241	10.0	Y	N	Nlp/P60 family
Rv1566c	RipD	Peptidoglycan binding	230	10.7	Y	Y (12, 13)	Nlp/P60 family
Rv1804c	Rv1804c	Conserved hypothetical protein	108	7.5	Y	Y (10, 12)	
Rv1926c	Mpt63	Secreted immunogenic protein/cell wall processes	230	10.7	Y	Y (10-12, 14, 15)	
Rv2297	Rv2297	Hypothetical protein	150	8.7	Y	Y (16)	
Rv2911	DacB2	Probable d-alanyl, d-ala carboxypeptidase	291	4.8	Y	Y (10, 11, 14)	Peptidase S11
Rv3269	Rv3269	Conserved hypothetical protein	93	7.8	N	Y (16)	
Rv3584	LpqE	Possible conserved lipoprotein	182	9.9	Y	Y (10, 11)	

Abbreviations: aa- amino acids, pI- isoelectric point, ss- predicted signal peptide sequence, Y- yes, N- no, CF- culture filtrate (in reference indicated in supplementary references)
