# Supplementary material for: Ubiquilin 1 Promotes IFN-γ-Induced Xenophagy of Mycobacterium tuberculosis
Source: PLoS Pathog. 2015 Jul 30;11(7):e1005076. doi: 10.1371/journal.ppat.1005076 (PMC4520715; doi:10.1371/journal.ppat.1005076)
Supplement: S1 Text — This file also contains the supplementary references. (DOCX) [file ppat.1005076.s009.docx]

**S1 Text**

**Supplementary Materials and Methods and Supplementary References**

**Yeast-two-hybrid (Y2H)**

Y2H assays were performed in strains Y8800 and Y8930 as previously described (1). MUPs were expressed as fusions to the Gal4-DNA binding domain and host proteins were fused to the Gal4–activation domain. Positive interactions result in growth of yeast on media lacking histidine with 5 mM 3-amino-1,2,4-triazole (3AT). Failure to grow in the presence of cyclohexamide was used to exclude autoactivators.

**Tissue culture**

RAW267.4 (RAW) and HEK293 cells were obtained from ATCC. They were grown in Dulbecco’s Modified Eagle Medium (DMEM; Gibco), 20 mM HEPES, 2 mM L-glutamine, and 10% heat inactivated fetal bovine serum (FBS; Life Technologies) at 37°C with 5% CO_2_. BMDMs were isolated and grown as previously described (2). Penicillin/Streptomycin (Gibco) was included except during infections. siRNAs were transfected with Hiperfect (Qiagen). Plasmids were transfected with Effectene (Qiagen). When bafilomycin A1 (Sigma) was used, it was added for 24 hours prior to cell lysis in PBS with 1% NP-40.

**Bacterial strains**

H37Rv was the wildtype *M. tuberculosis* strain. *ΔesxA* was provided by William Jacobs Jr. (Albert Einstein College of Medicine) (3). Δ*fbpB* was a gift from J. Ernst (NYU SOM). *M. tuberculosis* strains and *Mycobacterium smegmatis* mc^2^155 were grown at 37°C to log phase in Difco Middlebrook 7H9 media, 0.05% Tween 80, 10% BBL Middlebrook OADC Enrichment, and 0.2% glycerol. Plasmids were selected with 50 µg/µl kanamycin or hygromycin depending upon the resistance marker. Published plasmids were used for GFP expression (4) and for the live/dead analysis in Mtb (5). DsRed Mtb was a gift from J. Ernst (NYU SOM). The *Staphylococcus aureus* Newman strain was provided by V. Torres (NYU SOM) and grown to log phase at 37^o^C in RPMI media supplemented with casamino acids.

**Mice**

Atg16L1^flox/flox^-Lyz-Cre mice were described previously (6). Cre-negative Atg16L1^flox/flox^ littermates were used as controls. GFP-LC3 mice were provided by N. Mizushima (University of Tokyo). C57BL/6 and parkin (*Park2^tm1Shn^*) homozygous knockout mice were purchased from The Jackson Laboratory. P25 TCR-Tg mice were provided by J. Ernst (New York University School of Medicine; NYU SOM) (7). The New York University School of Medicine Institutional Animal Care and Use Committee approved all work with mice.

**Flow cytometry**

BMDMs treated with siRNAs and infected with Mtb at an MOI of 3 for 24h were fixed in 1% PFA and stained using Alexa Fluor 488 anti-mouse MHC class II. Flow cytometry was performed using a FACSCalibur at the NYU Cytometry Core.  FlowJo software was used for data analysis.

**Antigen Presentation**

BMDMs were treated with siRNAs 3d prior to infection and with IFN-γ 1d prior to infection. They were infected at an MOI of 5 and extensively washed at 4 hpi to remove extracellular bacteria. 24 hpi macrophages were incubated with P25 TCR-Tg CD4^+^ Th1 effector cells for 24h. P25 TCR-Tg CD4^+^ Th1 effector cells were generated *in vitro* as previously described (4). Briefly, CD4^+^ T cells were: (1) magnetically isolated from lymph node cell suspensions from P25 TCR-Tg mice using microbeads and an AutoMACS, (2) co-cultured with irradiated C57BL/6 splenocytes in the presence of mouse IL-12p70, IL-2, anti-IL4 neutralizing antibody, and synthetic peptide 25, and (3) washed with PBS, counted, and frozen until used. Culture supernatants from macrophage-T-cell co-cultures were collected after 24h and assayed for IFNγ by ELISA (BD Biosciences).

**Recombinant UBQLN1 protein production**

*E. coli* BL21 (DE3) cells were transformed with plasmids for expression of recombinant UBQLN1, UBQLN1 lacking the UBA domain (ΔUBA) or the UBL domain (ΔUBL) with an N-terminal GST-tag (8). Protein expression was performed in 2xYT media containing 100 μg/ml carbenicillin. Upon reaching an A_600_ 1.5 at 37°C, the protein expression was induced with 0.4 mM isopropyl β-D thiogalactopyranoside (IPTG) and the temperature was decreased to 30°C. After expression for 4h, the cells were pelleted, resuspended in lysis buffer (50 mM Tris-HCl pH7.7, 150 mM NaCl, 1 mM PMSF) and 3 U/mL Benzonase Nuclease (Novagen), 1 mg/ml of lysozyme (Sigma-Aldrich), and 5 mM EDTA were added. After incubation at room temperature for 40 min a centrifugation step at 12,000 g for 1h was carried out, and the cell-free supernatant was applied to a glutathione-Sepharose 4B column (GE Healthcare). The fusion proteins were eluted with lysis buffer containing 20 mM glutathione. Protein purity was evaluated by SDS-PAGE. Using Vivaspin filter with a molecular weight cut-off of 30,000 (Sartorius), the elution fractions were concentrated and the buffer was exchanged to PBS.

**Antibodies**

Three different UBQLN1 antibodies were used. The antibody used for co-immunoprecipitation, Abcam (ab3341), is a rabbit polyclonal raised to mouse Ubqln1 amino acids (aa) 2-18, which is 88% identical to human Ubqln1 and 47% identical to mouse Ubqln2. The same immunogen was used for the UBQLN1 antibody used for immunofluorescence (NB120-3341, Novus Biologicals), but no Freund’s adjuvant was used in its production. For western blots, we used Abnova mouse monoclonal UBQLN2 antibody, which was raised against aa 555-~624 of human Ublqn2, which is 93% and 91% identical to mouse Ubqln2 and mouse Ubqln1, respectively. The following additional primary antibodies were used: Myc (clone 9E10/sc-40, Santa Cruz Biotechnology), V5 (Invitrogen), Actin (clone C4/MAB1501, Millipore), FK2 (Millipore), p62 (ab56416, Abcam), MHCII (I-A/I-E, clone M5/114.15.2, Biolegend), parkin (ab15954, Abcam), UBQLN4 (ab106443, Abcam), and GST (Genscript). Secondary antibodies were: Alexa Fluor 594 goat anti-mouse, Alexa Fluor 555 goat anti-mouse, Alexa Fluor 647 goat anti-rabbit, HRP goat anti-mouse, and HRP goat anti-rabbit (Life Technologies).

**siRNAs**

The following siRNAs were used at 30 nM to silence UBQLN1: Thermo Scientific #D-041012-03 (siUbqln1 #1) and #D-041012-17 (siUbqln1 #2). Tsg101 (M-049922) siGENOME SMARTpool (Thermo Scientific) was used at 30 nM as previously described (2). siGENOME SMARTpool (Thermo Scientific) for UBQLN2 was (M-042279). ON-TARGET*plus* Non-Targeting siRNA #1 (Thermo Scientific) was used as a control.

**Plasmids**

Plasmids encoding MUPs, EsxA, EsxB, and Antigen 85b were provided by the Pathogen Functional Genomics Resource Center (PFGRC) as Gateway entry clones in pDONR221. They were then fully sequenced and subcloned into Y2H expression vectors. MUPs were cloned into pDEST40 for IP experiments. Plasmids containing human *UBQLN1* and human *NDP52* for Y2H experiments were obtained from the Center for Cancer Systems Biology Human ORFeome v5.1 collection (9) (<http://horfdb.dfci.harvard.edu/>) as Gateway entry vectors in pDONR223. These plasmids were then fully sequenced and subcloned into Y2H expression vectors. For expression of human UBQLN1 (isoform 1), UBQLN1-ΔUBA (amino acids 1-512), UBQLN1-ΔUBL (amino acids 131-589), and UBA (amino acids 540-589) in HEK293 cells, published myc-tagged and GST-tagged constructs were used (8). Murine Ubqln1, Ubqln2, and Ubqln4 were cloned from skeletal muscle cDNA (gift from S. Feske, NYU SOM) using the primers listed below into a Gateway entry vector and then moved into Y2H expression vectors. Ubqln4 was moved into pDEST40 for expression in HEK293 cells. The cDNA for murine Ndp52 was purchased from DNASU Plasmid Repository, transferred to the Gateway entry vector pDONR223 using primers indicated below, and subcloned into Y2H expression vectors.

| Gene  Name | Primers: (F- forward; R-reverse) |
| --- | --- |
| Ubiquilin 1  (variant 1) | F: GGGGACAACTTTTGTACAAAAAAGTTGGCACCATGGCCGAGAGCGCAGAG  R: GGGGACAACTTTTGTACAAAAAAGTTGGCAATGACGGCTGGGAGCCCAG |
| Ubiquilin1 (variant 2 ) | F: GGGGACAACTTTTGTACAAAAAAGTTGGCACCATGGCTGAGAACGGCGGAG  R: GGGGACAACTTTTGTACAAAAAAGTTGGCAAGGATGGCTGAGAGCCCAG |
| Ubiquilin 2 | F: GGGGACAACTTTTGTACAAAAAAGTTGGCACCATGGCCAAAGTGGAGAG  R: GGGGACAACTTTTGTACAAAAAAGTTGGCAAAGACTTCCTCAATTTCTCC |
| Ubiquilin 4 | F: GGGGACAACTTTTGTACAAAAAAGTTGGCACCATGGCGGAGCCGAGTG  R: GGGGACAACTTTTGTACAAAAAAGTTGGCAAGGAGAGCTGGGAGCC |
| NDP52  murine | F: GGGGACAACTTTGTACAAAAAAGTTGGCACCATGGACCAGTGCCCCATACCTACC  R: GGGGACAACTTTGTACAAGAAAGTTGGCAACCGGTGCAGATCATTCCACCAATAGG |

**Supplementary References**

1. Dreze M*, et al.* (2010) High-quality binary interactome mapping. *Methods Enzymol* 470:281-315.

2. Mehra A*, et al.* (2013) Mycobacterium tuberculosis Type VII Secreted Effector EsxH Targets Host ESCRT to Impair Trafficking. *PLoS Pathog* 9(10):e1003734.

3. Wong KW & Jacobs WR (2011) Critical role for NLRP3 in necrotic death triggered by Mycobacterium tuberculosis. *Cell Microbiol* 13(9):1371-1384.

4. Wolf AJ*, et al.* (2008) Initiation of the adaptive immune response to Mycobacterium tuberculosis depends on antigen production in the local lymph node, not the lungs. *J Exp Med* 205(1):105-115.

5. Martin CJ*, et al.* (2012) Efferocytosis is an innate antibacterial mechanism. *Cell Host Microbe* 12(3):289-300.

6. Marchiando AM*, et al.* (2013) A deficiency in the autophagy gene Atg16L1 enhances resistance to enteric bacterial infection. *Cell Host Microbe* 14(2):216-224.

7. Tamura T*, et al.* (2004) The role of antigenic peptide in CD4+ T helper phenotype development in a T cell receptor transgenic model. *Int Immunol* 16(12):1691-1699.

8. Kim SH*, et al.* (2009) Potentiation of amyotrophic lateral sclerosis (ALS)-associated TDP-43 aggregation by the proteasome-targeting factor, ubiquilin 1. *J Biol Chem* 284(12):8083-8092.

9. Lamesch P*, et al.* (2007) hORFeome v3.1: a resource of human open reading frames representing over 10,000 human genes. *Genomics* 89(3):307-315.
